# Site Specific Modification of Adeno-Associated Virus Enables Both Fluorescent Imaging of Viral Particles and Characterization of the Capsid Interactome

**DOI:** 10.1038/s41598-017-15255-2

**Published:** 2017-11-07

**Authors:** Jayanth S. Chandran, Paul S. Sharp, Evangelia Karyka, João Miguel da Conceição Aves-Cruzeiro, Ian Coldicott, Lydia Castelli, Guillaume Hautbergue, Mark O. Collins, Mimoun Azzouz

**Affiliations:** 1 Department of Neuroscience, Sheffield Institute for Translational Neuroscience (SITraN), The University of Sheffield, 385A Glossop Road, Sheffield, S10 2HQ UK; 2 Department of Psychology, The University of Sheffield, Sheffield, UK; 3 Department of Biomedical Science, The University of Sheffield, Sheffield, UK; 4 biOMICS Biological Mass Spectrometry Facility, The University of Sheffield, Sheffield, UK

## Abstract

Adeno-associated viruses (AAVs) are attractive gene therapy vectors due to their low toxicity, high stability, and rare integration into the host genome. Expressing ligands on the viral capsid can re-target AAVs to new cell types, but limited sites have been identified on the capsid that tolerate a peptide insertion. Here, we incorporated a site-specific tetracysteine sequence into the AAV serotype 9 (AAV9) capsid, to permit labelling of viral particles with either a fluorescent dye or biotin. We demonstrate that fluorescently labelled particles are detectable *in vitro*, and explore the utility of the method *in vivo* in mice with time-lapse imaging. We exploit the biotinylated viral particles to generate two distinct AAV interactomes, and identify several functional classes of proteins that are highly represented: actin/cytoskeletal protein binding, RNA binding, RNA splicing/processing, chromatin modifying, intracellular trafficking and RNA transport proteins. To examine the biological relevance of the capsid interactome, we modulated the expression of two proteins from the interactomes prior to AAV transduction. Blocking integrin αVβ6 receptor function reduced AAV9 transduction, while reducing histone deacetylase 4 (HDAC4) expression enhanced AAV transduction. Our method demonstrates a strategy for inserting motifs into the AAV capsid without compromising viral titer or infectivity.

## Introduction

Adeno-associated viruses (AAVs) are prominent gene therapy delivery vehicles increasingly used in clinical trials worldwide due to their low toxicity, ability to infect both dividing and post-mitotic cells, and long-lasting transgene expression^1,2^. A barrier to AAV use is the restricted cell and tissue tropism of many of the serotypes that limit their applicability to treating multisystem diseases. One option for overcoming this limitation is to select chimeric AAVs following a directed evolutionary approach with an affinity to a specific cell type, although the method does not clarify the identity of the receptor that the viral particle attaches to^3,4^. A second option, which has successfully re-targeted AAVs to muscle^5^ and endothelial cells^6,7^, is to present peptides onto the capsid surface with affinities for specific receptors^8^. Receptor-directed targeting of AAVs is an attractive option for tailoring the viruses to transduce specific cells, but to date only a few sites on the delicate network of proteins that form the structure of AAV particles appear to tolerate peptide insertions^8,9^.

AAVs house a genome containing a *cap* gene that is essential for capsid formation, yielding three structural proteins sharing a common C-terminus and differing through alternative splicing of the N-terminal region producing: VP1 at approximately 87 kDa, VP2 at 73 kDa, and VP3 at 62 kDa, with a molar ratio of 1:1:10.

Several reports have demonstrated that the loop region of AAVs exposed to the surface and defined by amino acids 584–590 in VP1 can tolerate peptide insertions without a significant loss of titer^7,10,11^. The same region has also been used to insert site specific sequences such as a biotin acceptor peptide recognized by a biotin ligase^10,12^, a small aldehyde tag that could be chemically synthesized to antibodies^13^, and unnatural amino acids that re-target the virus^14^. A comprehensive analysis of the AAV2 capsid suggested several sites on VP1 and VP2 (that differed from positions 584–590) capable of tolerating a human influenza hemagglutinin (HA) tag^9^, although viral titers were compromised by this alteration.

We sought to find a site different to the surface-exposed loop region that could tolerate the insertion of short peptides to increase the options for researchers designing AAV targeting experiments. We chose AAV9 as a good serotype to test optimal sites, since we could assess whether a short insert disrupted the ability of AAV9 to cross the blood brain barrier (BBB) and transduce cells in the central nervous system.

We inserted a 12 amino acid tetracysteine sequence described by Roger Tsien and colleagues^15^, which combines site specificity with low toxicity, into the AAV9 capsid at the VP1/VP2 interface (amino acid 138) after testing several different sites. Proteins expressing the core CCPGCC motif of the tetracysteine sequence can be labelled with fluorescently conjugated biarsenical dyes that selectively target reduced cysteine residues, and have been used to visualize the movement of bluetongue^16^ and alphaviral^17^ particles. Incorporating the tetracysteine sequence into this site had no effect on viral titer or infectivity, and did not compromise the ability of AAV9 to cross the BBB of mice.

We demonstrate the potential of this technology as a fluorescently labelled viral particle to examine viral particle dynamics, and use a biotinylated viral particle to characterize the capsid interactome using two different paradigms (1) whole cell analysis in HEK cells through direct labeling of newly formed AAV9 and (2) probing interactors in tissue by incubating biotinylated AAV9 capsids with mouse brain lysates. Toward validation of these interactomes, we assessed the biological relevance of two novel AAV9 interactors *in vivo*. αV and β6 subunits were identified as AAV9 interactors in HEK cells and we found that blocking the function of αVβ6 integrin led to a decrease in AAV9 transduction. Furthermore, reducing expression of histone deacetylase 4 (HDAC4), a multifunctional protein enriched in the brain^18^ which interacted with AAV9, enhanced AAV9 transduction. Our successful insertion of a site-specific sequence onto AAV9 provides a template for designing new tools to facilitate viral gene transfer and understanding AAV function.

## Results

### Identifying a site within the AAV9 capsid that tolerates a tetracysteine motif

All experiments in this study used a self-complementary AAV9 (scAAV9) driven by the cytomegalovirus (CMV) promoter expressing either GFP or mcherry.

**Figure 1:**
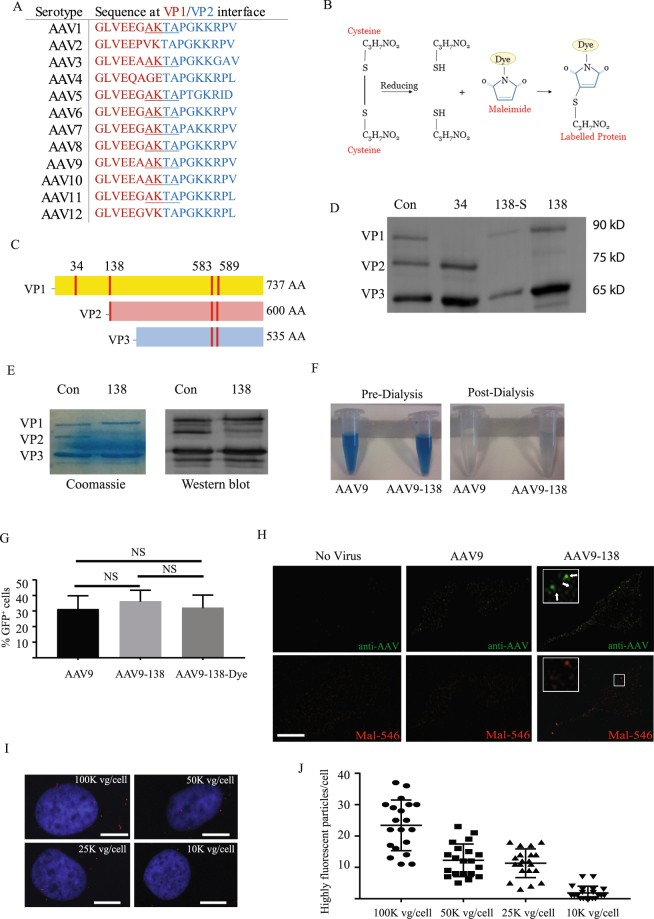
Insertion of tetracysteine into AAV9 capsid yields site specific labelling of viral particles. (**A**) The central four residues spanning the VP1/VP2 interface is conserved across many AAV serotypes; (**B**) A schematic for how maleimides recognize sulfhydryl groups on reduced cysteine residues resulting in labelled protein; (**C**) Tetracysteine sequences were cloned into four separate sites (red bars) that could either solely label VP1 (34), VP1 and VP2 (138), or all 3 capsid proteins (583, 589); (**D**) immunoblots probed with an antibody raised against all three AAV capsid proteins showed that the modification at amino acid 138, with the original alternate start codon (AAV9-138), was the only one to yield all three viral capsid proteins; (**E**) the tetracysteine sequence disrupted VP2 expression in AAV9-138 as illustrated by both Coomassie staining and immunoblotting of viral protein extracts with an anti-AAV antibody. (**F**) Dye labelled AAV9-138 retains a detectable level of the maleimide-660 dye in contrast to control AAV9 following dialysis; (**G**) There were no significant differences (Kruskal-Wallis test:p = 0.74) in the number of Hela cells transduced after 36 hours between the control AAV9, the AAV9-138, and the dye labelled AAV9-138 as determined by the number of GFP^+^ cells. Results are from three independent experiments, and cells were transduced with 100,000 vg/cell from each virus. (**H**) A subset of AAV9-138 particles labelled with a maleimide 546 co-localize with the viral particles detected by an anti-AAV antibody. Arrows point to particles that are detected by both the antibody and the maleimide-546 dye. Images shown are single planes taken with a Leica Sp5 confocal microscope. Scale bar, 10 µm. (**I**,**J**) Reducing the concentration of AAV9-138 results in a dose dependent decrease in high intensity fluorescently labelled particles. A maleimide-660-C2 dye was used for this experiment. Hoechst 33342 counterstained nuclei stained blue, scale bar, 10 µm.

An examination of the 12 major AAV serotypes showed that a core four residues (AKTA) were shared among nine of the serotypes and was therefore an insertion site which could be potentially applied to many different AAVs (Fig. 1A). Our design utilized the HRWCCPGCCKTF motif^15^ which exploits the specificity that thiol reactive compounds such as FlAsH/ReAsH^19^ and maleimides have for sulfhydryl groups that are generated from reducing cysteine disulfide bonds (Fig. 1B). We considered this motif a good candidate for selective labelling since the AAV9 capsid contains only four naturally occurring cysteine residues (C230, C291, C363, C396) that are not clustered together within the structure^20^, and are therefore unlikely to generate a strong signal relative to a tetracysteine tag. The three viral proteins (VP1, VP2, and VP3) that form the AAV capsid lattice differ at the N-terminus so that the entire VP3 sequence is contained within VP2, which is in turn contained within VP1 (Fig. 1C).

We initially tested the suitability of different sites (red bars, Fig. 1C) spanning the VP1 sequence that would lead to labelling of either solely VP1 (residue 34), VP1 or VP2 (residue 138), or all three capsids (residues 583, 589). These sites were chosen since they were either homologous to or proximal to residues on AAV capsids that other groups had demonstrated could withstand the epitope tag without compromising viral integrity^9–11^. Insertion of the tetracysteine motif at residue 34 of VP1 failed to generate an infectious virus, which was probably due to VP1, which is necessary for AAV capsid infectivity^21^, not being expressed (Fig. 1D). When we inserted the same motif after the alternate start codon at residue 138 that generates VP2 (AAV-138, Fig. 1D), we found that all three viral proteins were expressed, and the number of viral genomes was not significantly different to the unmodified AAV9 (AAV9 (n = 3): 3.5 × 10^13^ ± 1.3 × 10^13^ vg/ml; AAV9–138 (n = 9): 2.0 × 10^13 ± ^1.6 × 10^13^vg/ml, mean ± SEM, p = 0.46), although VP2 expression was faint (Fig. 1D). If we replaced the alternate start codon (ACG) with a standard ATG before the motif, we observed a ten-fold loss of viral protein concentration and nearly absent VP2 expression (AAV-138S, Fig. 1D). Both insertions of the tetracysteine motif into VP3 including the loop region (residues 583, 589) disrupted the formation of the capsid, and we were unable to detect any viral protein bands in a western blot. To determine whether the low VP2 expression in AAV9–138 could be due to a conformational change in the protein that compromised antibody detection, we compared viral proteins stained with a Coomassie stain (Instant Blue, Expedeon, UK) on a SDS-PAGE gel with the those immunoblotted with the same antibody. We observed a similar reduction in VP2 levels between the Coomassie stained gel and the western blot (Fig. 1E) for solely AAV9-138 confirming that the tetracysteine sequence disrupts VP2 expression.

### Fluorescently conjugated maleimides yield specific and robust labelling

We next examined whether the AAV9-138 virus could be labelled with a dye-conjugated thiol reactive maleimide to determine specificity and integrity of the reaction. We used maleimides as opposed to the FlAsH/ReAsH compounds first described with the tetracysteine sequence^15^, since it expanded the fluorescent spectra available for imaging, allowing us to avoid the higher sample background reported *in vivo* ^22,23^ with emission wavelengths between 520 nm–600 nm. For both the control AAV9 and AAV9-138, we reduced the cysteine disulfides with dithiothreitol (DTT, see materials and methods), and then incubated the viruses with an equimolar excess of fluorescent dye conjugated to maleimide ranging from 15-50 µM (Fig. 1F). When we dialyzed the viruses against PBS, we observed a detectable dye concentration only in AAV9-138 (n = 3; 1.5 ± 0.35 µM, Fig. 1F). Optimal dye concentration was 15 µM, and neither the tetracysteine sequence or the maleimide labelling method affected infectivity as demonstrated by quantifying GFP positive cells following a 36-hour transduction of Hela cells with an AAV9-GFP (Fig. 1G, AAV9: 31.0 ± 8.8, AAV9-138: 36.0 ± 7.4, AAV9-138-Dye: 31.9 ± 8.3, Kruskal-Wallis test: p = 0.74, Dunn Multiple Comparisons: each comparison p > 0.9; % GFP^+^ cells ± SEM). Fluorescent bands representing the viral proteins could not, however, be detected from dye labelled AAV9-138 run on an SDS-PAGE gel, suggesting that not all of the genetically modified capsids were efficiently labelled. To demonstrate that the dye labelling was specific to the viral capsid, we transduced Hela cells for 60 minutes with 100,000 vg/cell of labelled AAV9-138, and observed that a subset of fluorescently labelled viral particles co-localized with particles immunolabelled with an AAV antibody, with the difference likely attributed to the unlabelled VP3 protein. (Fig. 1H). We next determined the lower limit at which we could detect the viral particles with the strongest fluorescence *in vitro* in Hela cells after 60 minutes. We visualized a few intense viral particles at 10,000 vg/cell (Fig. 1I,J), but at this dilution found very few cells expressing a detectable GFP protein after a four-day transduction, so we opted for a dose of 50,000 vg/cell or higher at which Hela cells and other cell types tested showed a detectable GFP expression two days post transduction.

### Labelled viral particles are detectable *in vivo* and distribute into discrete areas

**Figure 2:**
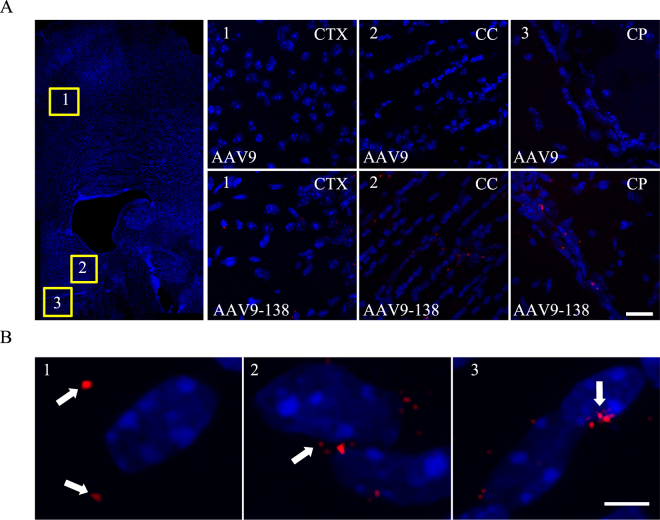
Viral particles are detected and distribute into distinct patterns *in vivo* (**A**) Confocal z-stack projections of images taken from coronal sections (40 µm) of adult mice (n = 3 mice were analyzed for both AAV9 and AAV9-138) show a clear fluorescent signal from the viral particles in the cortex (CTX), corpus callosum (CC) and caudate putamen (CP) two hours after intravenous delivery, Scale bar, 50 µm (**B**) Capsid proteins were distributed into three distinct patterns after two hours: (1) Away from the nucleus (25.7% ± 1.6%); (2) Perinuclear (66.1% ± 6.6%); (3) Intranuclear (2.1% ± 0.5%); 400 capsid puncta counted. Hoechst 33342 counterstained nuclei stained blue; Scale bar, 10 µm.

**Figure 3:**
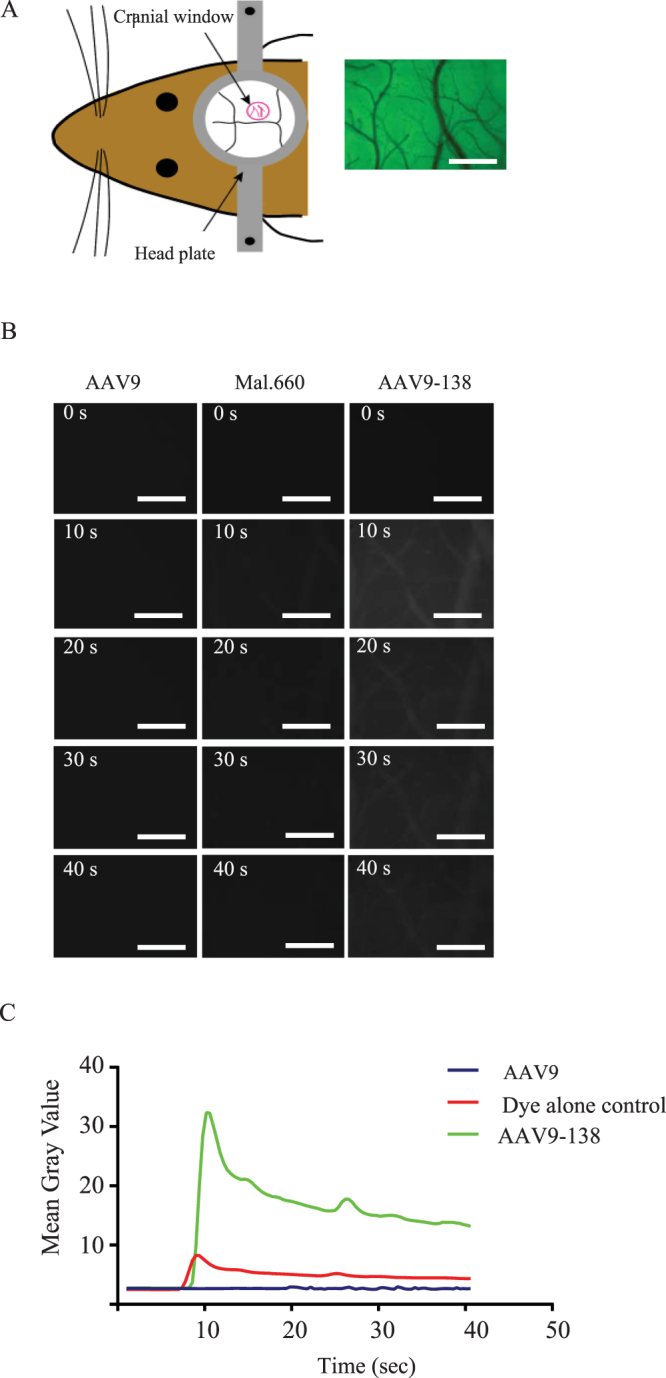
The fluorescent signal from viral particles is robust and remains visible in a cranial window more than 10 minutes after intravenous delivery to the tail vein of an anesthetized mouse. (**A**) A transparent cranial window at the parieto-occipital cortex of an adult mouse permits visualization of the pial vasculature; scale bar, 750 µm; (**B**) A bolus of AAV9-138-maleimide 660-C2 labelled viral particles remains visible in the cranial window after a tail vein injection unlike the dye control which washes out and the AAV9 control which has minimal fluorescence; scale bar, 375 µm; (**C**) The labelled AAV9-138 shows a much higher peak than the dye control or the control AAV9.

To determine whether the modified AAV9-138 retained the intrinsic property of AAV9 to cross the BBB^24–26^, we analysed adult mouse brains two hours after a single intravenous injection through the tail vein of 1.5 × 10^11^ vg of either AAV9 or AAV9-138. Both viruses were reduced with DTT, labelled with maleimide dye and dialyzed (see Materials and methods). We chose a short time point to limit viral diffusion that could dilute the signal. We were able to detect fluorescent viral particles in multiple regions of the CNS transduced with AAV9-138 (Fig. 2A), with the highest signal near the lateral ventricle, caudate putamen and cortex, and observed that they fell into three localization patterns (Fig. 2B): a minority (25.7% ± 1.6%) at a distance of between 10–20 µm from the nuclei (Fig. 2B–1); the majority (66.1% ± 6.6%) juxtapositioned around nuclei (Fig. 2B–2); and in rare cases (2.1% ± 0.5%) actually within the nuclei (Figs 2B–3).

To test whether AAV9-138 transduced the same number of cells in the CNS as AAV9 in mice, we injected 1.5 × 10^11^ vg of either AAV9-GFP or AAV9-138-GFP into P1 mice (Supplemental Fig. 1) through the facial vein and 12-week old adult mice through the tail vein (Supplemental Fig. 2) and sacrificed the mice two weeks later. We found no significant differences in neuronal (using a neuronal nuclei antibody NeuN) transduction between administration of AAV9-GFP and AAV-138-GFP in either early postnatal (Supplemental Fig. 1, AAV9 (n = 3): 84.0 ± 0.7, AAV9-138 (n = 4): 88.2 ± 3.3; % GFP^+^/NeuN^+^  ± SEM, p = 0.34) or adult mice (Supplemental Fig. 2, 100–150 GFP^+^ cells/mouse; AAV9 (n = 3): 95.7 ± 2.9, AAV9–138 (n = 3): 95.9 ± 2.5; % GFP^+^/NeuN^-^ ± SEM, p = 0.84). As previously reported^25^, both viruses transduced mostly neurons in the early postnatal mice and non-neuronal cells in adult mice and there were no significant differences between the viruses with respect to the number of GFP transduced cells across the brain in adult (AAV9 (n = 3): 3.2 ± 0.8, AAV9-138 (n = 3): 2.8 ± 0.6, p = 0.62, GFP^+^ cells/section, mean ± SEM) and P1 mice (AAV9 (n = 3): 42.2 ± 6.4, AAV9-138 (n = 3): 37.6 ± 5.6, p = 0.53, GFP^+^ cells/section ± SEM), (Supplemental Figs 1A, 2A). Taken together, AAV9-138 retains the ability to cross the BBB, and maintains a similar distribution of GFP transduction across the CNS as the AAV9 control.

### AAV9 capsids can be visualized *in vivo* with intravital imaging

To further explore the utility of the labelled virus, we assessed whether it could be visualized in real time *in vivo* following systemic delivery. Here our aim was to establish whether circulating viral particles could be visualized in the surface cerebral (pial) vasculature using conventional intravital microscopy, especially in light of the blood dilution effect following intravenous delivery. For real-time imaging of the pial vasculature, we used a thinned skull preparation, which consists of thinning the parietal bone until translucent to allow sufficient optical clarity (Fig. 3A). We began imaging prior to administering 1.5 × 10^11^ vg of AAV9-138 through the tail vein of an anesthetized mouse, and captured images every 400 ms for the duration of the experiment (Fig. 3B). We observed a wave of viral particles appearing as a bolus 9 seconds after injection, which reached a maxima between 10–15 seconds and then gradually decreased to a level above the background for 10 minutes (Fig. 3B,C). When we compared this profile to injections of a dye control we noted that the dye control has a smaller peak at a similar time to the labelled virus, but then rapidly washes out. This suggests that a significant quantity of virus remains associated with the vasculature after a systemic injection in a healthy adult mouse and confirms that fluorescently conjugated AAV9-138 has a robust and identifiable signal *in vivo*.

### Biotinylation of viral particles yield capsid interactome

**Figure 4:**
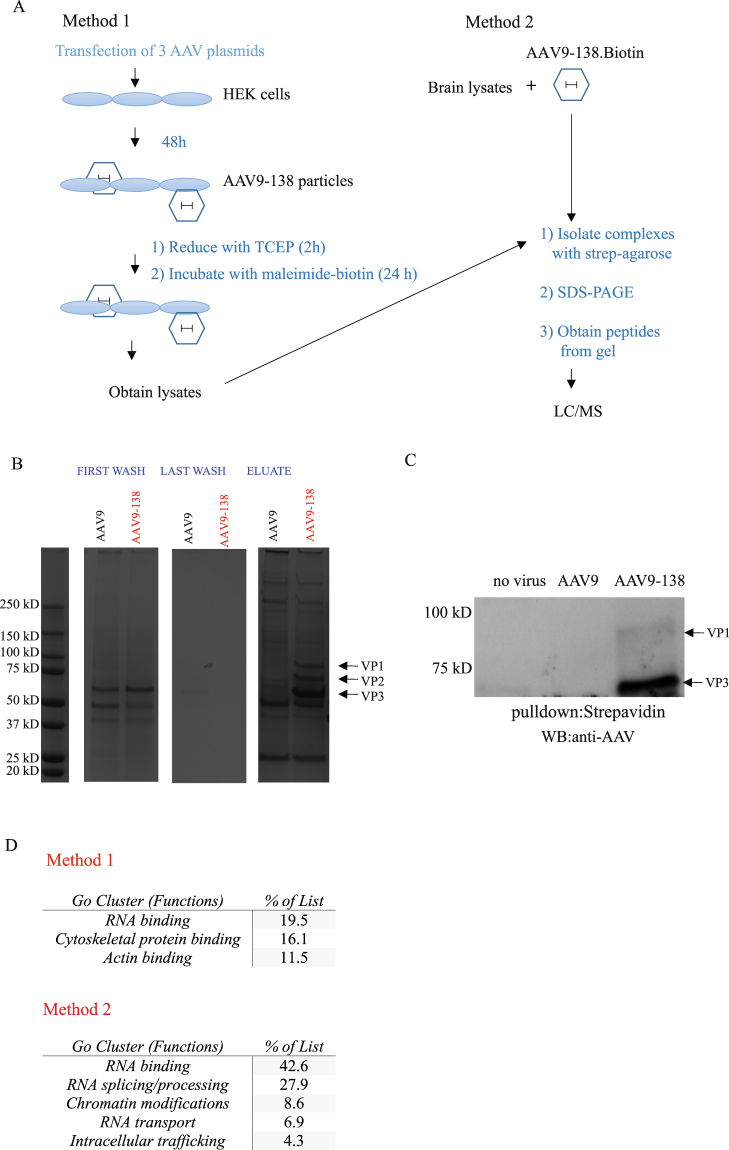
Biotinylation of modified AAV9 capsid enables the identification of interacting proteins. (**A**) Schematic showing two different ways to identify AAV9 interactors; In method 1, AAV9 particles are labelled in HEK cells 48 hrs after being generated before the complexes are isolated with streptavidin-agarose and identified through LC/MS; In method 2, purified high-titer AAV9 particles which have been biotinylated are mixed with mouse brain lysate before pulldowns (**B**) Instant Blue stained gel with fractions from pulldown with method 2 show enrichment of virus in AAV9-138 lane only; FW = first wash; LW = last wash. (**C**) Immunoblotting of streptavidin pulldowns with an anti-AAV antibody reveal bands corresponding to VP1 and VP3 in the AAV9-138 that are absent in controls (**D**) Gene ontology (GO) analysis taken from functional protein interaction networks (http://string-db.org/) reveals importance of RNA-binding and the actin cytoskeleton.

We next explored whether AAV-138 particles could yield molecular clues about the basic biology of AAV infection. To achieve this, we biotinylated the viral capsid with a maleimide-linked biotin compound and purified protein complexes binding to the virus with streptavidin agarose. We used two different approaches (Fig. 4A) to compile a comprehensive data set of the AAV interactome, and expand the range of experimental paradigms that could utilize our labelling strategy. In the first method, we generated either control AAV9 or AAV9-138 particles in HEK293T cells following our standard three plasmid transfection protocol (see materials and methods), and labelled the capsids directly in the cells with a maleimide-biotin. We then prepared whole cell lysates from the labelled cells, and used streptavidin-agarose to pull down AAV9 protein complexes. We reasoned that this method would test the feasibility of obtaining AAV interactors in a simple tractable system such as a cell line, which are amenable to simple chemical and genetic manipulations and can therefore be used to answer a unique question. We also hypothesized that labelling newly formed AAV9 particles directly in HEK293T cells by adding maleimide-biotin to the culture media would enable us to enrich for viral interactors at the cell surface due to the poor membrane permeability of maleimide-biotin^27,28^. In contrast, for method 2 (Fig. 4A), we incubated labelled and purified AAV9 particles with adult mouse brain lysates to get a more comprehensive interactome, as many proteins are down-regulated during passaging of immortalized cell lines compared to primary cells^29^, and due to the importance of AAV9 in CNS related therapies^30^. We were able to use a stringent set of wash buffers for both methods due to the strong affinity of biotin for streptavidin, resulting in minimal background in the last wash fractions of the immunoprecipitation, and a clear and unambiguous detection of all three viral capsid protein bands in AAV9-138 that was clearly absent in control AAV9 (Fig. 4B). The presence of all three viral capsids in the eluate shows that VP1 and VP2 associate with VP3 which lacked the tetracysteine sequence. We were surprised, however, to see that the concentration of VP2 appeared to be similar to VP1 in the biotinylated AAV9-138 in Fig. 4B, when we had previously observed that VP2 expression was diminished following insertion of the tetracysteine sequence (Fig. 1D,E). To clarify this discrepancy, we immunoblotted the streptavidin pulldowns with an anti-AAV antibody, and once again noted only bands corresponding to VP1 and VP3 (Fig. 4C). From this, we conclude that the band appearing between VP1 and VP3 in Fig. 4B is probably not VP2, but a different protein of similar molecular weight.

When we examined the peptides from the pulldowns with LC/MS using the first method, we identified 67 proteins either unique to AAV9-138 or at a five-fold excess to AAV9 (Supplemental Table 1). The three major functional clusters associated with these interacting proteins were RNA binding, cytoskeletal protein binding and actin binding proteins (Fig. 4D). We found a much larger list of proteins using the second method probably due to the larger diversity and abundance of proteins in whole mouse brain, and refined it to 335 proteins (Supplemental Table 2) by including two criteria: (1) at least two unique peptides; (2) unique to the AAV9-138, or at a five-fold excess compared to AAV9 (see Materials and methods).

A comparison of gene ontology (GO) analysis from three different online databases (http://string-db.org/; www.reactome.org; https://david.ncifcrf.gov/home.jsp) again highlighted RNA binding as a highly represented function of proteins in the dataset (Fig. 4D).

**Figure 5:**
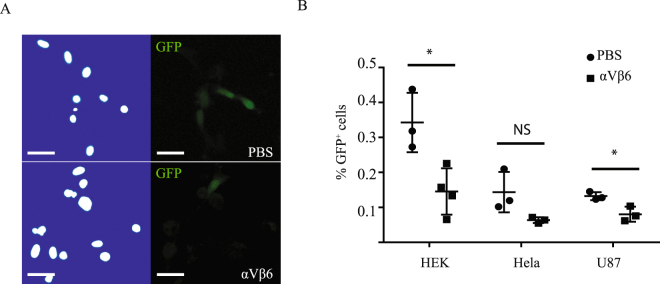
Blocking integrin receptor αVβ6 reduces AAV9 transduction in multiple cell lines. (**A**) In U87 cells, pretreatment with 20 µg/ml of mouse anti-human αVβ6 integrin for 2 h reduces AAV9-GFP transduction as measured after 24 h compared to a PBS control. (**B**) Similar reductions in AAV9-GFP transduction are observed in HEK293T (n = 4), Hela (n = 4) and U87 (n = 3) cells that are pretreated with the αVβ6 integrin functional blocking antibody compared to PBS controls. Error bars represent mean ± SEM. **p < 0.01; ***p < 0.001; Scale bar 20 µm. Student’s unpaired *t*-test was used to compare the two groups. Hoechst 33342 counterstained nuclei stained white on a blue background.

To validate the two datasets, we decided to examine the relationship between proteins from each interactome and AAV9 transduction. Previous reports have shown that the integrin αVβ5 and α5β1 heterodimers can act as AAV co-receptors and mediate viral entry into the cell^31–33^. As alpha-V (ITGAV) and integrin beta-6 (ITGB6) were detected in the dataset from ‘method 1’, we decided to test the effects of blocking the αVβ6 heterodimer with a functional blocking antibody in several different cell lines that were permissive to AAV transduction. Pre-treatment of either HEK293T, Hela or U87 cells with 20 µg/ml of a mouse anti-human αVβ6 antibody significantly reduced AAV9-GFP transduction by nearly 50% (Fig. 5A,B) confirming a role for αVβ6 integrin in mediating AAV9 transduction.

**Figure 6:**
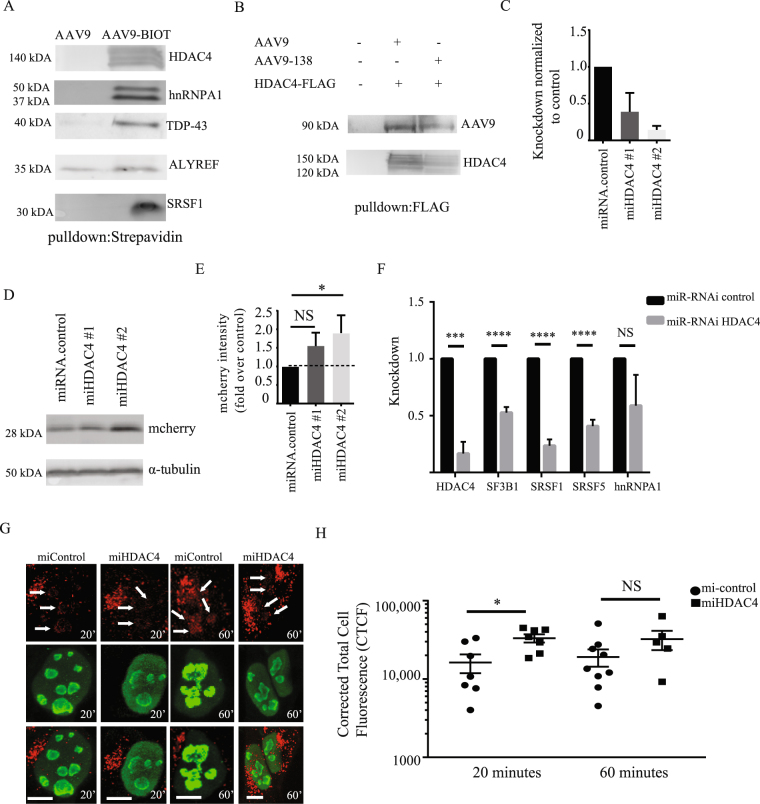
Modulating HDAC4 expression enhances AAV9 transduction. (**A**) Western blot analysis of pulldowns between whole brain lysates and AAV9 (4 × 10^11^ vg for either virus) with streptavidin-agarose corroborates data from LC/MS. Note that endogenous SRSF1 expression was not detectable by conventional western blot, and so SRSF1-flag was overexpressed in HEK cells and incubated with the AAVs. Images shown are from multiple blots taken from a single pulldown probed for different interactors. Pulldowns were repeated at least three times for all targets shown. (**B**) Both AAV9 and AAV9-138 can bind to HDAC4 indicating that the HDAC4-AAV interaction does not arise purely from the capsid modification. Images shown are the same pulldown probed on different blots. (**C**) Two different miRNA sequences for HDAC4 demonstrate at least a 50% knockdown in HEK293T cells normalized to the control. (**D**) Western blot analysis of protein lysates taken 48 hrs after AAV-mcherry was added at 50,000 vg/cell show that the miHDAC4 #2 sequence augmented mcherry expression compared to a control miRNA scramble sequence. Tubulin immunoreactivity measured after stripping blot of mcherry signal. (**E**) Densitometry of western blots show that pretreating cells with the miHDAC4 #2 sequence yielded a 1.9 ± 0.48 (n = 5) fold increase in mcherry expression. (**F**) HDAC4 knockdown significantly (Student’s unpaired *t*-test used for each gene) reduces SF3B1, SRSF1, and SRSF5 levels in HEK293T cells stably expressing either a lentiviral miR-RNAi control or a miR-RNAi-HDAC4. (**G**,**H**) HDAC4 knockdown HEK cells show a significant increase in AAV9 particles in the nucleus compared to miR-RNAi controls at 20 minutes (Mann-Whitney test, n = 7, *p = 0.026) but not 60 minutes (Mann-Whitney test, miControl: n = 9; miHDAC4: n = 5, p = 0.15). Corrected total cell fluorescence (CTCF) was used to quantify the AAV9-138 particles as described in methods. Arrows indicate regions where viral particles are clearly co-localizing with nucleolin. Error bars represent mean ± SEM. The AAV9-138 particles are stained red and nucleolin is stained in green. Scale bar 10 µm.

We decided to validate the second dataset by repeating the pulldown experiments and immunoblotting for several proteins that were within the 335 protein interactome and spanned different functional classes (Fig. 4D), as well as assessing proteins excluded from the dataset due to either not having two unique peptides or being below the 5-fold cut-off. Proteins in the interactome, such as heterogeneous nuclear ribonucleoprotein A1 (hnRNPA1), an RNA-binding protein involved in pre-mRNA processing^34,35^, HDAC4, which is important in chromatin remodelling^36^ and transactive response DNA binding protein 43 kDa (TDP-43), which is an RNA and DNA-binding protein^37^ were validated after immunoblotting biotinylated AAV9-138 complexes (Fig. 6A).

We also examined splicing factor 2/alternative splicing factor SRSF1, which was excluded from the dataset since only 1 peptide was detected despite being unique to AAV9-138, and ALYREF, which was excluded since it was below the 5-fold excess cut-off. We specifically chose these two proteins since we have previously observed that both proteins have roles in RNA binding, splicing, and export^38–40^, which are functional categories overrepresented in our dataset (Fig. 4D) and because many of our AAV9-138 interactors are present in the SRSF1 interactome^35^. We were unable to either detect endogenous SRSF1 in mouse brain lysates or pulldown endogenous SRSF1 in the mouse brain lysates with our biotinylated AAV-138, suggesting that expression of the protein in brain was below the limits of antibody detection. To test this, we mixed lysates from HEK293T cells transfected with a full length SRSF1 fused to a FLAG tag with AAV9 (1 × 10^12^ vg) or AAV9-138 (1 × 10^12^ vg), pulled down the complex with streptavidin agarose and detected SRSF1 in an immunoblot (Fig. 6A). When we probed the whole brain-AAV9-138 complex for ALYREF, however, there were bands in the immunoblots in both control AAV9 and AAV9-138, supporting our 5-fold exclusion limit (Fig. 5A). Collectively, these results set the thresholds of the interactome, although they may exclude low-abundance proteins.

### Disruption of HDAC4 enhances AAV transduction

We hypothesized that HDAC4 may act as a barrier of AAV transduction as part of a stimuli/stress response based on published data^41,42^, and tested this by reducing its expression in HEK293T cells with a miR-RNAi-GFP before transducing the cells with an AAV9-mcherry. We first checked whether binding of HDAC4 to AAV9 occurred in an unmodified AAV9 by incubating the control AAV9 (1 × 10^12^ vg) with HEK293 protein lysates overexpressing HDAC4-FLAG, and immunoprecipitating the complexes with anti-FLAG-agarose. We could clearly see that HDAC4 binds to conventional AAV9 and AAV9-138 (Fig. 6B), and that the HDAC4 association with AAV9 was not a new association created by the tetracysteine tag.

We then tested the efficacy of two different miR-RNAi sequences in knocking down *HDAC4*, and found that both sequences knocked down at least 50% of the expression, with miHDAC#2 showing the strongest reduction (Fig. 6C, miHDAC4#1 (n = 3):0.39 ± 0.20; miHDAC4#2 (n = 3):0.14 ± 0.05; mean ± SEM). When we knocked down protein expression with each miR-RNAi sequence before infecting cells with AAV9-mcherry, we found that depletion of HDAC4 with both miR-RNAi sequences enhanced mcherry expression (Fig. 6D,E, fold change relative to control: miHDAC4#2 (n = 5): 1.6 ± 0.43; miHDAC4#2 (n = 5): 1.9 ± 0.48; mean ± SEM), demonstrating that HDAC4 can inhibit AAV transduction.

Due to the large number of spliceosomal proteins detected in our interactome, which have been shown previously can inhibit AAV transduction^43^, we examined whether a functional relationship exists between spliceosomal genes and HDAC4. We stably knocked down HDAC4 in HEK293T cells by transducing the cells with a lentivirus expressing either a miR-RNAi control or miR-RNAi-HDAC4 sequence, and consistently observed a knockdown of at least 80% (Fig. 6F). When we examined the expression of spliceosomal genes encoding proteins in our interactome dataset in these stable HEK293T lines, we found that HDAC4 knockdown significantly suppressed SRSF1, SRSF5 and SF3B1 in HEK293T cells compared to miR-RNAi controls suggesting a role for HDAC4 in maintaining spliceosomal expression (Fig. 6F).

We hypothesized that one by-product of HDAC4 inhibition would be enhanced AAV migration to the nucleolus, where viral transduction is influenced by interactions with nucleolin^44,45^. We added AAV9-138 at 50,000 vg/cell to the stable HEK293T control or HDAC4 knockdown lines, and examined intracellular viral particle distribution after 20 and 60 minutes. Viral particles co-localized with nucleolin (arrows, Fig. 6G) within 20 minutes in both control and HDAC4 knockdown cells and continued to accumulate in the nucleolus at 60 minutes. The number of particles associating in and around the nucleus was significantly higher (p = 0.014) in the miHDAC4 HEK293T cells compared to controls at 20 minutes, but not at 60 minutes (p = 0.21), suggesting that HDAC4 can inhibit the first wave of AAV9 particles to the nucleus (Fig. 6H).

## Discussion

We describe here a method for site-specific labelling of the AAV9 capsid using a tetracysteine motif. This alteration in the capsid enabled us to visualize the fate of viral particles in cells and generate a capsid interactome without sacrificing titer or infectivity or compromising the ability of AAV9 to cross the blood-brain barrier. To our knowledge, this is the first study to generate an unbiased dataset of AAV interactors using an intact and infectious virus as bait, and we expect this data to be useful to the gene therapy community.

Our site of integration of the tetracysteine sequence into the AAV9 capsid at the VP1/VP2 interface (amino acid 138) was based on previously published data with the AAV2 capsid that showed sites capable of tolerating epitope tags^9,10^. The insertion of a motif into VP3 of AAV9 failed to generate an infectious virus, and as a result, our labelling strategy does not account for the VP3 capsid, which is in molar excess. As the VP1 and VP2 proteins in AAV2 are biased in their localization towards the nucleus compared to VP3^46^, it is probable that our AAV9-138 detects fewer cytoplasmic particles than are actually present. Interestingly, there appears to be either a size or residue charge specific limit that can be incorporated into the VP1/VP2 interface of the AAV9 capsid; we could generate infectious AAV9 particles following a 14 amino acid V5 epitope (GKPIPNPLLGLDST) insertion, but could not with a 22 amino acid 3xFLAG motif (DYKDHDGDYKDHDIDYKDDDDK)(unpublished observations, JSC). We would favour a residue specific limitation on the placement of epitopes into the AAV capsid since both a 13 amino acid aldehyde tag^13^ and a 23 amino acid biotin acceptor peptide^10^ were inserted into the AAV2 capsid without compromising viral integrity, though the insertional sites differed from the one used in our study.

Long-term *in vivo* imaging in rodents using intravital microscopy has greatly improved our understanding of the natural dynamics of biological processes^47^. Currently, it remains unclear exactly what is happening to the viruses *in vivo* after being administered due to the difficulty in observing these events in real time. To take a step towards addressing this limitation, we measured the fluorescent signal of our labelled viral particles in an anesthetized adult mouse using a cranial window (Fig. 3A) that enabled us to image surface pial vessels at depths of 50 µm. At this depth, we were able to visualize a rapid bolus of labelled AAV9-138 virus appearing within 9 seconds of intravenous delivery (Fig. 3B). Our labelling method provides a platform for setting up a similar system in combination with higher resolution two-photon microscopy, to improve signal and extend the depths of that tissue that can be imaged.

There have previously been two distinct approaches taken to classify the molecules mediating AAV transduction: yeast two-hybrid screens using portions of the capsid as bait^48^ and siRNA library screens that assess transduction after disrupting gene expression^43,49,50^. The interactomes we identified in HEK cells (Supplemental Table 1) and whole mouse brain lysates (Supplemental Table 2) have minimal overlap with the genes recovered from the yeast two-hybrid screen using mouse liver cDNA as a library, though the authors identified nucleotide binding and intracellular trafficking as key functional categories of AAV interactors, which pathway analysis of our data also identified (Fig. 4D). There are several plausible reasons for the differences in interactomes: tissue type, AAV serotype, and our use of an intact virus as opposed to a capsid fragment. The inference from both sets of data is that the AAVs bind to a unique set of proteins depending on tissue type, though that will need to be experimentally verified in future studies.

We used two different labelling strategies to get a more comprehensive interactome and to also setup a platform for designing downstream experiments. Direct labelling of newly generated, unpurified AAV9 particles in HEK293T cells yields more temporal control than simply incubating lysates with a purified biotinylated AAV9, since conditions can be manipulated *in vitro* before and after biotinylation to allow specific questions to be tested. The drawback to this approach is that many proteins are not expressed as highly in cell lines compared to primary cells^29^, so it is likely that a significant number of proteins will be excluded from analysis due to poor detection. This may, in addition to the different tissue of origin, account for why the interactomes from the two different methodologies we used largely differ, though ontology analysis highlights the importance RNA-binding proteins in both datasets.

We were intrigued by the enrichment of spliceosome proteins in our LC/MS analysis from the mouse brain dataset, and a recent siRNA screen delineated a role for the U2 snRNP spliceosome complex in restricting AAV9 transduction^43^, and showed an interaction between the splicing factor SF3B1 and the AAV9 particles, which we also detected (Supplemental Table 2). Our data suggests that the importance of spliceosomal machinery in inhibiting AAV transduction is not, however, restricted to the U2 snRNP complex, as we detected more than 10 other proteins not linked to the U2 snRNP complex that co-purify with the spliceosome^51^. An independent whole genome siRNA screen^50^ detected an abundance of genes linked to DNA damage and repair, which we noted in our dataset (Supplemental Table 2) with both HDAC4^52,53^ and RNA-binding proteins and splicing factors which all have functions linked to DNA damage^54–56^. We focused on HDAC4 for further analysis due to its putative neuroprotective function and role in the DNA damage response^52,57^, and since data suggests that HDAC inhibition enhances AAV transduction^58^. We found that HDAC4 knockdown enhanced AAV9 transduction and suppressed the expression of several snRNP genes including SF3B1, SRSF1 and SRSF5 (Fig. 6F). Whether the loss of snRNP expression explains why HDAC4 inhibition improves AAV9 transduction will need to be experimentally verified in the future.

In conclusion, we have genetically modified the AAV capsid to permit a range of functional analyses including intravital imaging of viral particles and proteomic analysis of the capsid interactome. Although our preliminary analysis suggests that the introduction of the modification at residue 138 does not affect the infectivity or distribution of AAV9 in the CNS, further tests will need to be performed to check whether the AAV9-138 and AAV9 interactomes are similar. We consider that our experimental approach could be applicable to other serotypes and potentially used to enhance our understanding of current AAV-based gene therapy applications.

## Materials and Methods

### Antibodies and Reagents

Chicken anti-GFP (1:1000, Abcam), Rabbit anti-GFP (1:5000, Invitrogen), mouse anti-hnRNP-A1 (1:1000, Millipore, clone 9H10), Rabbit anti-Nucleolin (1:2000, Abcam, ab22758), Rabbit anti-HDAC4 (1:1000, Abcam, 12172), Mouse anti-TDP43 (1:1000, Abcam, 57105), Mouse anti-γH2AX-pS139 (1:1000, Abcam, 26350), Mouse anti-FLAG (1:2000, Sigma, F3165), Rabbit anti-AAV VP1, VP2, VP3 (1:1000 (WB), 1:50 (ICC), #03-61084, American Res Prod, Waltham, MA), Mouse anti-Integrin αVβ6 (Millipore, MAB2077Z) and mouse anti-NeuN (1:300, Millipore) were used at the dilutions listed above. Alexa Fluor dye (546, 660-C2) maleimide dye (Life Techologies) and Biotin-maleimide (B1267, Sigma) was maintained in aliquots at a stock concentration of 10 mM in DMSO at −20 °C. LC/MS grade acetonitrile (Pierce), ammonium bicarbonate (Sigma), and Pierce™ Trypsin Protease, MS Grade (90057), and formic acid (Sigma) were used for LC/MS described below.

### Cell lines used

The U87 glioblastoma cell line was a kind gift from Spencer Collis at the University of Sheffield, and HEK 293 T and HeLa cells were purchased from the American Type Cell Collection (ATCC). All cell lines were maintained in growth media (GM) consisting of Dulbecco’s modified Eagle’s medium (DMEM, D5796, Sigma Aldrich) supplemented with 10% fetal bovine serum (Sigma Aldrich), penicillin (100 U/ml), and streptomycin (100 U/ml) at 37 °C and 5% CO_2_.

### Generation of AAV9-138 capsids

The scAAV9-GFP plasmids were received from St. Jude Children’s Research Hospital (Memphis, TN). The tetracysteine motif HRWCCPGCCKTF was embedded into a forward primer as an inverse PCR reaction (F, AAV9-138: 5′-CATCGATGGTGTTGCCCGGGCTGCTGTAAG

ACTTTCGCTCCTGGAAAGAAGAGGCCTGTAG-3′; R, AAV-138: 5′-CGTCTTAGCCGCTTCCTCAACCAG-3′), using Phusion high-fidelity polymerase (New England Biolabs, USA) to amplify the changes made to the pAAV2/9 plasmid containing the AAV9 capsid sequence. To initially test whether the modified capsids were capable of generating an AAV9 virus, one 15 cm dish containing HEK293T cells at 80% confluence was transfected with 30 µg total DNA using polyethylenimine (PEI; MW ~ 25 K) with a mixture of three plasmids (with 15 µg: 7.5 µg: 7.5 µg in order listed) that are required to generate an infectious AAV9 viral particle: (1) a plasmid providing helper genes isolated from adenovirus that enhance viral infectivity (pHelper); (2) an ITR-containing plasmid carrying the gene of interest (pAV2-CMV-GFP); (3) a plasmid that carries the AAV Rep-Cap proteins (pAAV2/9); For all of the experiments described in this manuscript, we used the pAV2-CMV-GFP consisting of two ITRs in a truncated genome that resulted in a self-complementary AAV9 (scAAV9), as described by others^59^. Two days following transfection, cell lysates were pelleted, resuspended in PBS, and the virus was extracted from the cells by three cycles of rapid freezing on dry ice followed by thawing at 37 °C. Following the second freeze/thaw, the viral protein lysate was passed through a 21-gauge needle five times to enhance viral recovery. Following the final thawing of the lysate, the viral supernatant was collected after a 10-minute spin at 12,000 g/4 °C, incubated with 100 units of benzonase for 1 hour, passed through a 0.22 µm filter, and stored in aliquots.

### Construction of miR-RNAi vectors

To express artificial microRNAs (miRNAs), we used a BLOCK-iT Pol II miR RNAi system (Invitrogen), and followed the protocol explicitly. The human miRNA sequences were chosen from the RNAi designer (Invitrogen) with only ‘5 star’ sequences chosen, and then annealed into duplexes and inserted into the linearized miRNA expression vector pcDNA6.2-GW/ EmGFP-miR according to the manufacturer’s protocol (Invitrogen). For *HDAC4*, sequence #2 was a chained set of two different miRNAs (following the manufacturer’s protocol) including the one used in sequence #1.

Two miRNA hairpins were designed against human *HDAC4* (GenBank: NM_006037.3):

Sequence#1 (start:566): top strand (mature miR-RNAi sequence in blue)

5′TGCTGAAATGCAGTGGTTCAGATTCCGTTTTGGCCACTGACTGACGGAATCTGCCACTGCATTT-3′

Sequence#1:bottom strand (complement in red)

5′CCTGAAATGCAGTGGCAGATTCCGTCAGTCAGTGGCCAAAACGGAATCTGAACCACTGCATTTC -3′

Sequence#2 (start:732): top strand (mature miR-RNAi sequence in blue)

5′TGCTGTTCAGATTCGGTTCAGAAGCTGTTTTGGCCACTGACTGACAGCTTCTGCCGAATCTGAA -3′

Sequence#2:bottom strand (complement in red)

5′CCTGTTCAGATTCGGCAGAAGCTGTCAGTCAGTGGCCAAAACAGCTTCTGAACCGAATCTGAAC -3′.

### Large scale AAV9 production and site-specific labelling

Sixty 15 cm plates containing HEK293T cells at 80% confluence were transfected using PEI as described in the previous section to generate AAV9 particles. Four days after transfection, the AAV9 enriched media was collected, incubated at 37 °C for 2 hours with 3,750 units of benzonase-nuclease (Sigma Aldrich), filtered through a 0.22 µm filter, and concentrated to 1 ml using Amicon spin filter units (Millipore). The virus was then washed with 50 ml of phosphate buffered saline (PBS, pH 7.3) in the same Amicon spin filter units, and concentrated to a final volume of 1 ml. The viral sample volume was expanded to 14 ml with PBS and separated through a discontinous iodixanol (D1556, Sigma Aldrich) gradient (4 ml of 54%, 9 ml of 40%, 9 ml of 25%, 5 ml of 15%), and centrifuged at 69,000 rpm for 1.5 hours at 18 °C. The purified virus, which was found as a white layer between the 54% and 40% iodixanol gradient was subsequently removed in 0.5 ml fractions using a syringe, and 10 µl of each fraction was mixed at an equal ratio with a 2X reducing sample SDS-PAGE buffer, heated to 75 °C for 20 minutes, separated on a 4–20% precast TGX mini-gel (Biorad), and stained with Sypro-Ruby according to the manufacturer’s protocol (Life Techologies). Fractions that showed a pure virus composed solely of the VP1, VP2 and VP3 bands were combined, and washed against 5 full volumes (15 ml each) of PBS with an Amicon spin filter, before collecting in a final volume of between 300–500 µl. This purified virus was reduced in 10 mM Dithiothreitol (DTT)/PBS for 2 h at 4 °C with gentle agitation, and the DTT was desalted out by adding 60 ml of PBS (7.3), and spinning through an Amicon spin filter to a final volume of 1 ml. The reduced virus was then incubated with 15–50 µM of the maleimide labelling dye (or maleimide-biotin), with DTT added to a final concentration of 5 µM, and labelling proceeded for 24–48 hrs with gentle agitation at 4 °C. The labelled virus was subsequently dialyzed with a slide-a-lyzer dialysis cassette (ThermoScientific) against PBS (pH 7.3) to remove unbound dye, and the concentration of the dye was measured using a Nanodrop ND-1000 (ThermoScientific).

### Lentiviral production

The miR RNAi construct was sub-cloned into a self-inactivating lentiviral vector (SIN-W-PGK)^60^, which was a gift from Dr Nicole Deglon (Lausanne, Switzerland), using standard cloning methods. Twenty 10 cm dishes seeded with 3 × 10^6^ HEK293T cells/dish were each transfected with 13 μg pCMVΔR8.92, 3.75 μg pM2G, 3 μg pRSV and 13 μg SIN-CMV-miRNA with PEI. Media was replaced after 12 hours, and then 48 hours later, supernatant was collected, filtered through a 0.45 μm filter and centrifuged at 19,000 rpm for 90 minutes at 4 °C. The supernatant was discarded and the viral pellet was resuspended in 1% BSA in PBS and stored at −80 °C. The biological titre of the viral vector was determined by transducing HeLa cells with 10^−2^, 10^−3^ and 10^−4^ dilutions of the vector. 72 hours post-transduction, cells were fixed in 4% paraformaldehyde, washed in PBS, and the percentage of GFP positive cells was measured with a fluorescent-activated cell sorter (FACS, LSRII). The biological titer, expressed as the number of transducing units per ml (TU/ml), was calculated with the following formula: Vector titer = [(% positive cells × number of cells during transduction) × dilution factor × 2] TU/ml.

### Generation of HDAC4 knockdown HEK293T lines

HEK293T cells were transduced with lentivirus expressing either miR-RNAi-control or miR-RNAi-HDAC4 at a MOI of 5. Five days after transduction, cells were passaged every three days. For all the experiments in this manuscript, HDAC4 knockdown was assessed at each passage by qRT-PCR as described elsewhere in the methods.

### Whole Brain Pulldowns

Whole brain lysates were prepared by homogenizing freshly dissected tissue from 2-month-old male C57/bl6 mice in a ‘L1’ lysis buffer (2 ml per brain; 50 mM Tris-HCL, pH 7.5, 150 mM NaCl, 1% NP-40 (Generon), 0.5% sodium deoxycholate (Sigma) with a protease inhibitor cocktail (PIC; Sigma, P8340)) with 12 strokes from a pre-chilled Dounce homogenizer. The protein extract was further lysed by agitating at 4 °C for 30 minutes, and then passed through a 21-gauge needle to shear nucleic acids. Extracts were pelleted by centrifuging at 17,000 g for 4 °C for 10 minutes, and the supernatant was removed for use in the IPs. Strepavidin-agarose slurry (25 µl agarose slurry per IP; GE Healthcare Life Sciences, 17-5113-01) was washed once with PBS and resuspended in 1 ml of 1% BSA/PBS for 2 hours at 4 °C with agitation. The beads were then washed twice with PBS, before incubating at 4 °C with agitation for 1 hour with either a control AAV9 (1 × 10^12^ vg) or the biotinylated AAV9-138 (1 × 10^12^ vg). Two milligrams of whole brain supernatant were then added to each pulldown, and gently agitated overnight at 4 °C. Beads were washed 4 times with a wash buffer (PBS + 1% NP-40), followed by two washes with lysis buffer, before being resuspended in 2X Laemmli buffer with 5% β-mercaptoethanol and boiled for 5 minutes to elute the bound proteins.

### Direct Maleimide-Biotinylation of AAV9 in HEK cells

Two 15 cm plates containing HEK293T cells at 80% confluence were transfected using PEI as described in the previous section to generate AAV particles. Two days after transfection, proteins were reduced for two hours with 10 mM Tris(2-carboxyethyl) phosphine hydrochloride (TCEP, Sigma #646547) solution diluted in GM, washed once with PBS, and biotinylated with 20 µM maleimide-biotin diluted in GM for 24 hours. Cells were washed twice with PBS, before resuspending with ice cold L1 buffer and breaking cells open with 10 strokes from a dounce homogenizer and then agitating for 30 minutes at 4 °C. Cell suspensions were centrifuged at 20,000 g at 4 °C for 10 minutes. The supernatant (S1) was removed and placed into a fresh tube, and the crude membrane pellet was resuspended in ice-cold L1 buffer with 10 strokes from a dounce homogenizer and agitated for a further 20 minutes. Cellular debris was pelleted by centrifuging at 2,000 g at 4 °C for 10 minutes, and the supernatant was combined with the previous S1 pool to generate the lysate used for pulldowns. Strepavidin-agarose slurry (25 µl agarose slurry per IP) was washed once with PBS and resuspended in 1 ml of 1% BSA/PBS for 2 hours at 4 °C with agitation. The beads were then washed twice with PBS, before incubating at 4 °C overnight with 2 mg total of either maleimide-biotin treated control HEK lysate (AAV9 transfected) or AAV9-138 transfected lysate. Beads were washed 4 times with a wash buffer (PBS + 1% NP-40), followed by two washes with lysis buffer, before being resuspended in 2X Laemmli buffer with 5% β-mercaptoethanol and boiled for 5 minutes to elute the bound proteins.

### FLAG-pulldowns

HDAC4-FLAG^61^ (gift from Eric Verdin; Addgene plasmid #13821) was transfected into HEK293 cells with PEI, and the cells were collected 2 days later, washed with PBS, and lysed in a hypotonic buffer (10 mM Hepes, pH 7.9, 1.5 mM MgCl_2_, 10 mM KCl, 0.5 mM DTT (Sigma), with PIC) at 4 °C with agitation for 1 hour. Extracts were pelleted by centrifuging at 17,000 g/10 min at 4 °C, and the supernatant was retained for use in the pulldown. M2-FLAG-agarose slurry (25 µl agarose slurry per IP; Sigma, A2220) was washed once with PBS before the beads were blocked with 1% BSA for 2 hours at 4 °C with agitation. The beads were then washed twice with PBS, before incubating at 4 °C with agitation for 1 hour with two milligrams of the HEK lysate containing overexpressed HDAC4-FLAG, before AAV9 (1 × 10^12^ vg) or AAV9-138-Biotin (1 × 10^12^ vg) was then added to each pulldown, and gently agitated overnight at 4 °C. Beads were washed 6 times with a wash buffer (PBS + 1% NP-40), before being resuspended in 2X Laemmli buffer with 5% β-mercaptoethanol and boiled for 5 minutes to elute the bound proteins.

### Immunoblotting

Protein (and crude viral lysates) were mixed at an equal ratio with a 2X reducing sample SDS-PAGE buffer, heated to 75 °C for 20 minutes, separated on a 4–20% precast TGX mini-gel (Biorad), and transferred onto a PVDF membrane (Millipore). Membranes were blocked with 5% BSA in TBS with 0.05% Tween (TBST) for 30 minutes, before incubating at 4 °C overnight with agitation with the appropriate antibody diluted in 5% BSA in TBST. Following three washes in TBST for 10 minutes each, membranes were incubated at room temperature with mild agitation with an appropriate secondary HRP antibody (Sigma Aldrich, 1:5000) diluted in 5% BSA in TBST, washed three times with TBST for 10 minutes each, and developed with enhanced chemiluminescent substrate (Amersham-Pharmacia Biotech, USA).

### Proteomic analysis of viral particle interactomes

Eluted mouse brain proteins from AAV and AAV-138 viral particle purifications were separated using SDS-PAGE with a 4–20% precast TGX mini-gel (Biorad), and visualized with Instant Blue (Expedeon, Cambridge, UK), and the entire lane was cut into 4 equal fractions. In-gel digestion was performed as reported previously^62^. Extracted peptides were analysed by nanoflow LC-MS/MS using an Orbitrap Elite (Thermo Fisher) hybrid mass spectrometer equipped with a nanospray source, coupled to an Ultimate RSLCnano LC System (Dionex). The system was controlled by Xcalibur 2.1 (Thermo Fisher) and DCMSLink 2.08 (Dionex). Peptides were desalted on-line using a micro-Precolumn cartridge (C18 Pepmap 100, LC Packings) and then separated using a 60 min RP gradient (4–32% acetonitrile/0.1% formic acid) on an EASY-Spray column, 15 cm × 50 µm ID, PepMap C18, 2 µm particles, 100 Å pore size (Thermo). The LTQ-Orbitrap Elite was operated with a cycle of one MS (in the Orbitrap) acquired at a resolution of 60,000 at m/z 400, with the top 20 most abundant multiply-charged (2 + and higher) ions in a given chromatographic window subjected to MS/MS fragmentation in the linear ion trap. An FTMS target values of 1e6 and an ion trap MSn target value of 1e4 was used and with the lock mass (445.120025) enabled. Maximum FTMS scan accumulation time of 500 ms and maximum ion trap MSn scan accumulation time of 100 ms were used. Dynamic exclusion was enabled with a repeat duration of 45 s with an exclusion list of 500 and exclusion duration of 30 s. MS data was analysed data using MaxQuant^63^ version 1.5.2.8. Data was searched against mouse UniProt sequence databases (downloaded June 2015) using following search parameters: trypsin with a maximum of 2 missed cleavages, 7 ppm for MS mass tolerance, 0.5 Da for MS/MS mass tolerance, with Acetyl (Protein N-term) and Oxidation (M) set as variable modifications and carbamidomethyl (C) as a fixed modification. A protein FDR of 0.01 and a peptide FDR of 0.01 were used for identification level cut offs. The dataset was filtered to remove proteins with less than 2 unique peptides in AAV9-138 purifications giving a dataset of 974 proteins. Label free quantification was performed using MaxQuant calculated protein intensities^64^. AAV-138 enriched proteins were defined as those that were specific to the AAV9-138 purification and those that had a AAV9-138/AAV9 ratio > 5, giving a final set of 335 AAV9-138 enriched proteins.

### Viral genome counts using quantitative PCR

All reactions were done using the Quantifast SyBR Green PCR Kit (Qiagen, Cat 204054) on a BioRad CFX96 thermal cycler, following the manufacturer’s instructions. The number of GFP copies in three dilutions of a purified AAV9 virus (100x, 1000x, 10,000x) was compared to a standard curve generated by serial dilutions of a linearized pAV2-CMV-GFP vector. Primers used to quantify viral genomes were (Poly A, Forward: 5′-ATT TTA TGT TTC AGG TTC AGG GGG AGG TG-3′), (PolyA, Reverse: 5′-GCG CAG AGA GGG AGT GGA CTA GT-3′), (GFP, Forward: 5′- GAC GGC AAC ATC CTG GGG CAC AAG-3′), and (GFP, Reverse: 5′: CGG CGG CGG TCA CGA ACT C-3′).

### Mcherry expression following miR-RNAi knockdown

5 × 10^4^ HEK293T cells were plated per well of a 12 well plate 18–24 hours before transfecting with the appropriate miR-RNAi scramble or HDAC4 miR-RNAi target sequence. All the transfections had a similar efficiency (~90% cells transfected), which we determined with GFP that was expressed in the miR-RNAi plasmid. Three days later, AAV9-mcherry was added at 50,000 vg/cell to each well, and the virus was given 24 hours to transduce cells, before cells were washed with PBS, and lysed in ice cold L1 buffer for thirty minutes on ice, passed through a 23-gauge needle to shear genomic fragments, and pelleted by centrifugation at 17,000 g × 10 min at 4 °C. The supernatant was collected, measured by a BCA protein assay (Pierce), and 10 µg of total protein was used as described in ‘immunoblotting’.

### Timecourse of AAV9 distribution

For assessing AAV9 distribution in HEK293T cells stably expressing either a miR-RNAi-control or miR-RNAi-HDAC4 from a lentivirus, purified AAV9-138 was added to cells at 50,000 vg/cell and fixed with 4% PFA after 20 minutes and 1 hour. Confocal stacks of the nuclear focal planes were assembled, and the viral particles associating with the nucleus were quantified using ImageJ. The viral particles were quantified using a corrected total cell fluorescence (CTCF) measurement (described in https://sciencetechblog.com/2011/05/24/measuring-cell-fluorescence-using-imagej) and utilized in Burgess *et al*. (2010)^65^. Briefly, the CTCF defines the fluorescence detected per cell with the integrated density parameter on Image J, and incorporates the background fluorescence into the measurement.

### Integrin Blocking Assay

HEK293T, Hela or U87 cell lines grown to 60% confluency in a 96-well plate (Greiner) were pretreated with 20 µg/ml (in GM) of mouse anti-human integrin αVβ6 antibody for 2 hours at 37 °C before 50,000 vg/cell of AAV9-GFP diluted in an equal volume of GM was added and incubated at 37 °C for 24 hours. Cells were rinsed once with PBS and fixed in ice-cold 4% paraformaldehyde for 8 minutes at room temperature, washed three times for 10 minutes with PBS before counterstaining with 5 µg/ml of Hoecsht 33342 (Sigma Aldrich) at 5 µg/ml for 15 minutes. The nuclei and the GFP^+^ cells were imaged in the 96-well plate with an IN Cell Analyzer (GE Healthcare Life Sciences) with 10 random fields chosen per well, and the average taken from two wells. Three to four biological replicates were performed per cell line.

## Ethics Statement

All animal procedures were performed in compliance with UK Home Office regulations (Scientific Procedures Act, 1986) through project license 40/3739 (held by MA) and approved by the University of Sheffield Ethical review committee. Mice were housed in a 12 hour light/dark cycle and fed a regular diet *ad libitum*.

### AAV *in vivo* administration for immunohistochemistry

For examining the transduction capability of the modified and control AAV9, postnatal day 1 (P1) C57BL/6JRcc strain (Harlan, Bicester, UK) mice were injected with a single intravenous injection of 1.5 × 10^11^ vg via the facial vein, sacrificed two weeks later with a lethal overdose of Euthatal, and transcardially perfused with cold 4% paraformaldehyde (PFA) in 0.1 M phosphate buffer. Brains were dissected and postfixed overnight at 4 °C in PFA, transferred to 30% sucrose for 48 hours, embedded in OCT compound (Cell Path, UK), and snap frozen in an isopentane/dry ice bath. Brains were cut in 40 μm thick sagittal sections on a cryostat (Leica), placed directly onto charged slides, and then air dried. Every 6^th^ section across the brain was processed for immunocytochemistry. Sections were incubated with gentle agitation for 2 hours at room temperature in blocking buffer (PBS with 5% normal goat serum, 3% BSA and 0.2% Triton-X-100) before incubating overnight at 4 °C with a chicken anti-GFP antibody and mouse anti-NeuN diluted in PBS with 3% BSA. Following three washes with PBS, sections were incubated with a goat anti-rabbit Alexa-488 conjugated secondary antibody (Invitrogen, CA) for 1 hour at room temperature in 3% BSA, counterstained with Hoechst 33342 and the signal was protected with Fluoromount (Sigma-Aldrich).

### AAV *in vivo* administration for assessing maleimide-labelled viral capsids

Adult (8-week old, bodyweight 23 g) C57BL/6JRcc strain (Harlan, Bicester, UK) mice were injected with a single intravenous injection of 5 × 10^10^ vg of either AAV9 or AAV-138 via the tail vein, and sacrificed two hours later with a lethal overdose of Euthatal, and transcardially perfused with cold 4% paraformaldehyde (PFA) in 0.1 M phosphate buffer. Brains were dissected and postfixed overnight at 4 °C in PFA, transferred to 30% sucrose for 48 hours, embedded in OCT compound (Cell Path, UK), and snap frozen in an isopentane/dry ice bath. Brains were cut in 40 μm thick sagittal sections on a cryostat (Leica), placed directly onto charged slides, and then air dried. Sections were blocked in 1% BSA for 1 hour, washed twice with PBS, counterstained with Hoechst 33342 and mounted with Fluoromount. Sections were assessed spanning the brain to determine where the signal was highest, and in these sections (largely comprising the caudate putamen, corpus callosum and cortex), every 5^th^ section was analysed for the fluorescent signal.

### Intravital imaging

An adult female C57BL/6 mouse (23 g) was anaesthetised with fentanyl-fluanisone (Hypnorm, Vetapharm Ltd), midazolam (Hypnovel, Roche Ltd) and water (1:1:2 by volume; 1.0 ml/kg, intraperitoneal) for surgery. The mouse was loaded into stereotactic frame complete with a gas anaesthesia head holder (Kopf; 923-B) and a homoeothermic blanket (Harvard Apparatus) to maintain rectal temperature at 37 °C. To form a cranial window, the scalp was removed and a circular area of parietal bone (~2 mm in diameter) was carefully thinned using a high-speed dental drill until the pial vasculature was clearly visible. A thin layer of clear cyanoacrylate cement was applied to reinforce and smooth the window. At least two hours was allowed between the completion of surgery and imaging. For imaging, we used a fluorescence stereomicroscope (Leica M205FA) with a 20.5:1 motorized zoom function, a 2.0x PlanApo lens, which can achieve a magnification of 32x and a resolution of 0.95 µm. The tail vein was cannulated (29 G needle/PE10 polyethylene tubing) for intravenous infusion of fluorescently labelled virus and the plasma marker (FITC-labelled 2MDa dextran; Sigma FD2000s). To visualise surface cerebral vasculature the cranial window was imaged using a band pass filter for GFP (Em 525/50 M) and excitation of 470/40 nm. Here, we used intrinsic tissue autofluorescence as contrast for the less fluorescent vasculature. The plasma marker was not used initially to identify blood vessels to prevent potential binding of the virus with circulating high MW dextrans. To image circulating labelled virus we used a narrow band pass filter (690/50 M) and excitation of 640/30 nm. Circulating virus was imaged for 4.5 mins at ~2.5 f/s. At the end of the imaging experiment (>1 h after virus infusion), 2MDa FITC-dextran (2 mg in 100 µl saline) was administered intravenously to assess stability of the vascular network.

### Statistical analysis

Results are shown as mean ± standard error (S.E.M.). Student’s unpaired *t*-test for two group comparisons was used to analyse viral genomes, labelling efficiency, and effects of the αVβ6 blocking antibody on AAV9 transduction. The Mann-Whitney test was used to analyse viral capsids in stably transduced HEK293T cell lines. A Kruskal-Wallis test was used to test analyse GFP transduction differences between the control AAV9, the AAV9-138 and the AAV9-138-dye viruses. For all tests, a *P*-value < 0.05 was taken as statistically significant, and Graphpad Prism 6 was used for all statistical analyses.

### Data availability

All data generated or analysed during this study are included in this published article (and its Supplementary files).

## Electronic supplementary material

Supplemental Information

Table S1

Table S2
