# Supplementary material for: Site Specific Modification of Adeno-Associated Virus Enables Both Fluorescent Imaging of Viral Particles and Characterization of the Capsid Interactome
Source: Sci Rep. 2017 Nov 7;7:14766. doi: 10.1038/s41598-017-15255-2 (PMC5676692; doi:10.1038/s41598-017-15255-2)
Supplement: Supplementary file 1 — Supplemental Information [file 41598_2017_15255_MOESM1_ESM.pdf]

Supplemental Information

Site Specific Modification of Adeno-Associated Virus Enables Both Fluorescent Imaging of Viral Particles and Characterization of the Capsid Interactome

Jayanth S. Chandran[1], Paul S. Sharp[1,2], Evangelia Karyka[1], João Miguel da Conceição Aves-Cruzeiro[1], Ian Coldicott[1], Lydia Castelli[1], Guillaume Hautbergue[1], Mark O. Collins[3,4], and Mimoun Azzouz[1*]

Figure S1

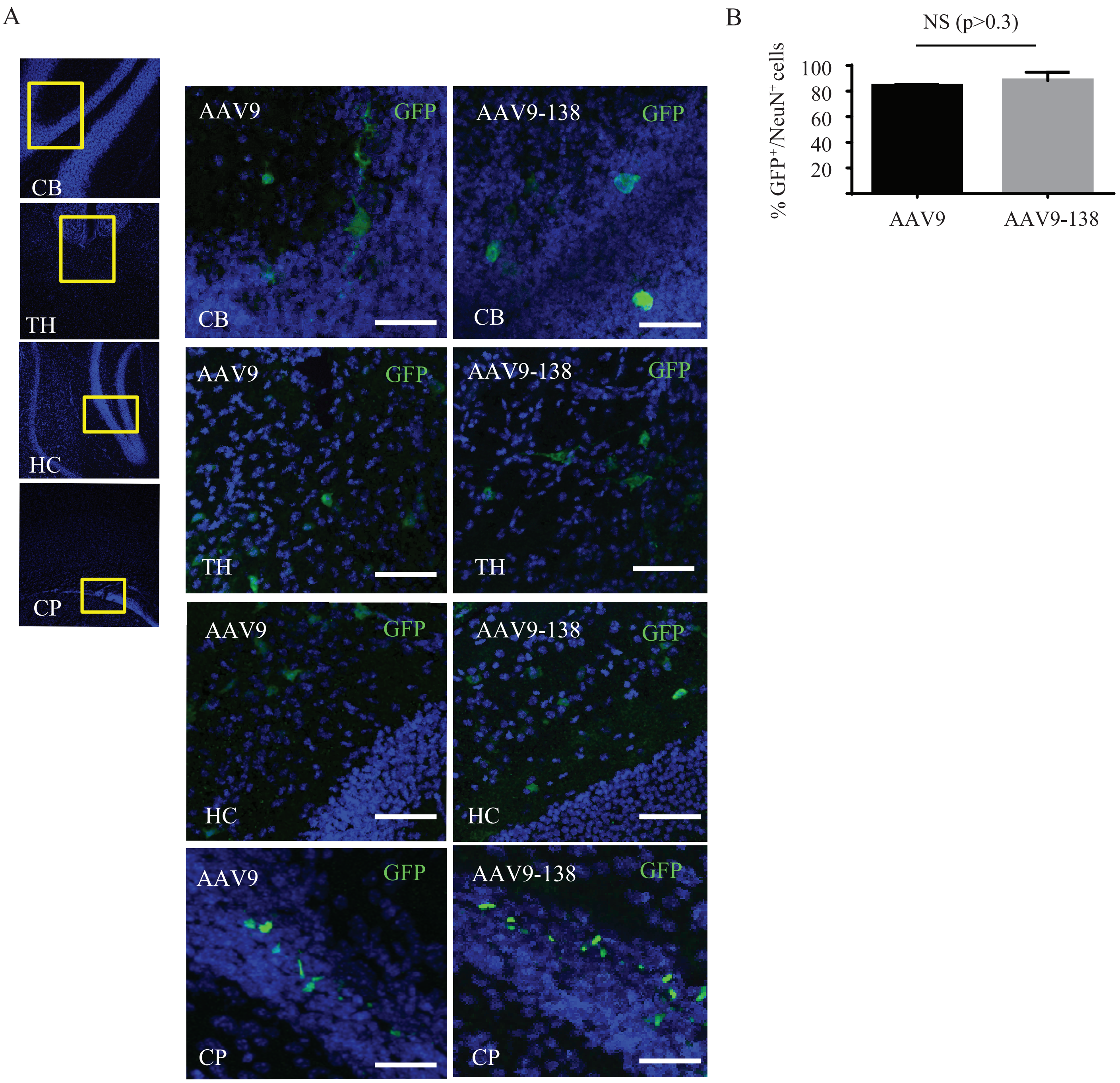

Supplemental Figure 1. AAV9-138 biodistribution in early postnatal mice is similar to unmodified AAV9 following facial vein delivery. (A) Similar distribution of GFP transduction was observed in the brains of P1 mice given a single facial vein injection of 1.5x1011 vg of either control AAV9 or AAV9-138 virus, and then sacrificed two weeks later; scale bar 100µm. (B) GFP transduction occurred largely in NeuN+ neurons irrespective of which virus was injected; AAV9 (n=3): 84.0 ± 0.7, AAV9-138 (n=4): 88.2 ± 3.3; % GFP+/NeuN+ ± SEM, Student's unpaired t-test :p=0.34.

Figure S2

A

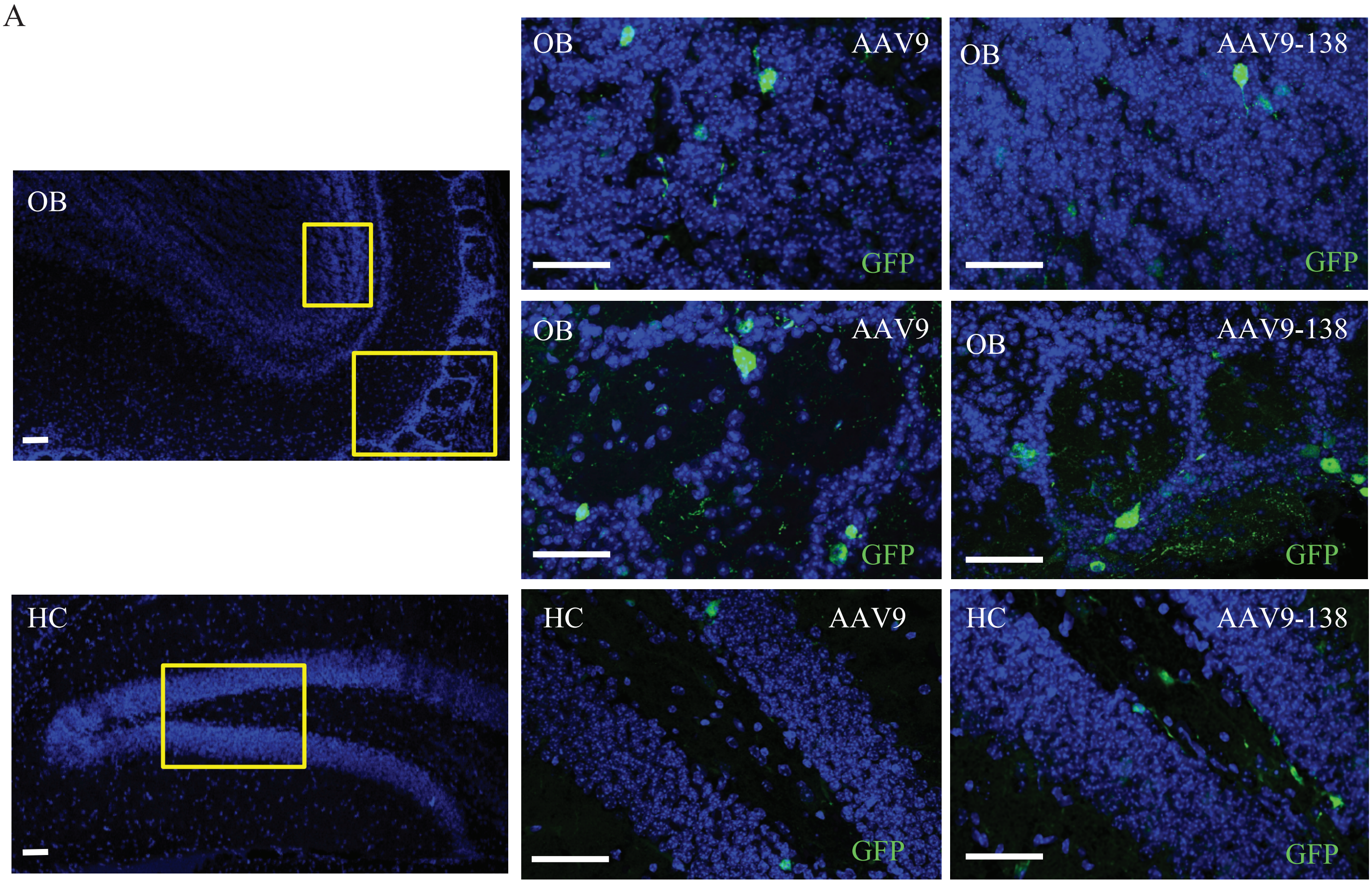

B

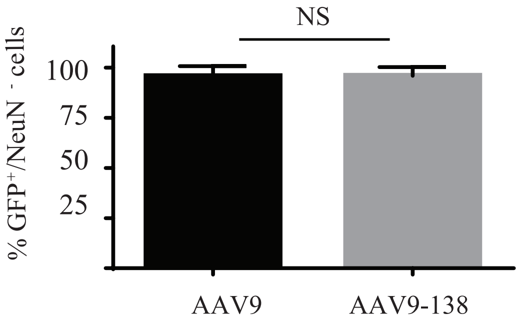

Supplemental Figure 2. AAV9-138 biodistribution in adult mice is similar to unmodified AAV9 following tail vein delivery. (A) A single injection of 1.5x1011 vg of either AAV9-GFP or AAV9-138-GFP through the tail vein of adult mice revealed a similarly sparse GFP transduction across the brain with enrichments at the olfactory bulb and hippocampus; scale bar 100µm. (B) Cell tropism was not altered in the AAV-138 as the majority of GFP+ transduced cells following tail vein injection of virus in the adult mice were non-neuronal (100-150 GFP+ cells/mouse; AAV9 (n=3): 95.7 ± 2.9, AAV9-138 (n=3): 95.9 ± 2.5; % GFP+/NeuN- ± SEM, Student's unpaired t-test :p=0.84).

Figure 1D. Uncropped image

MW Marker

1

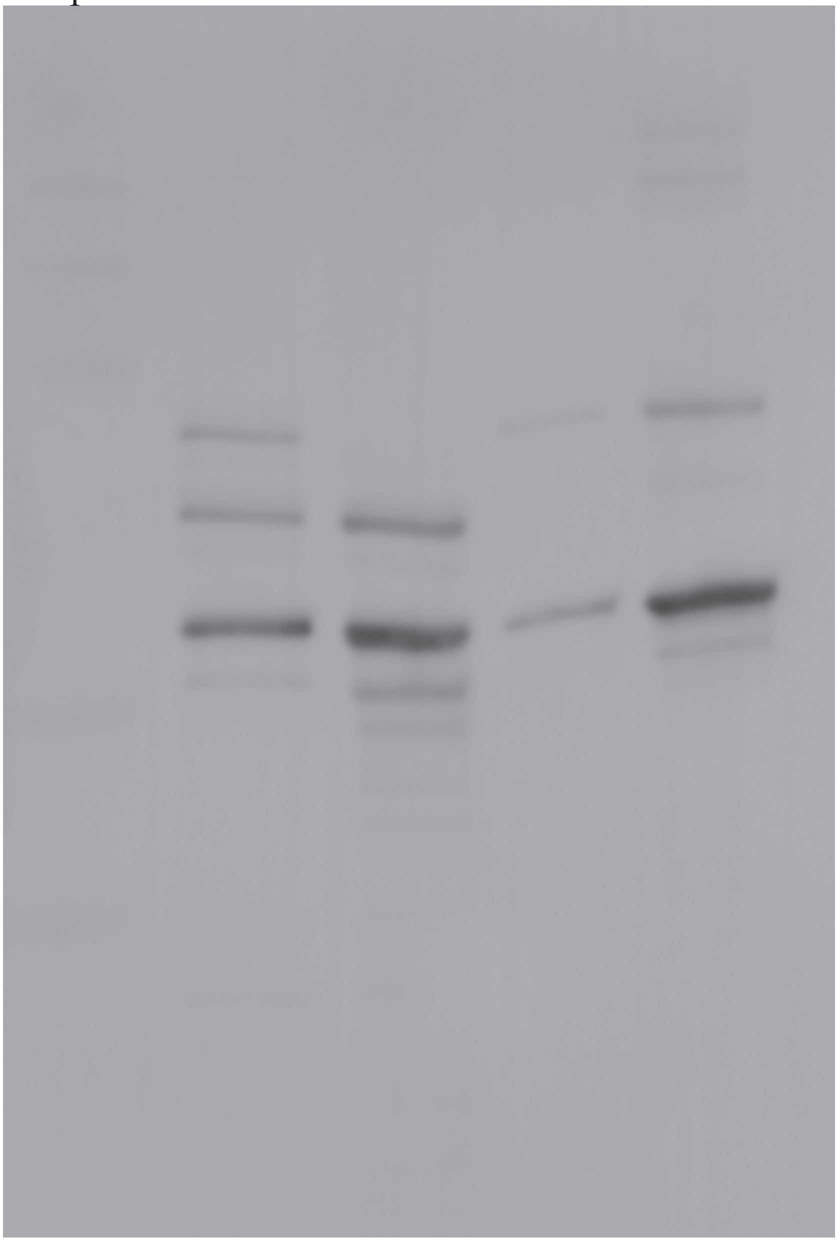

Figure 4C. Uncropped image

MW Marker

1

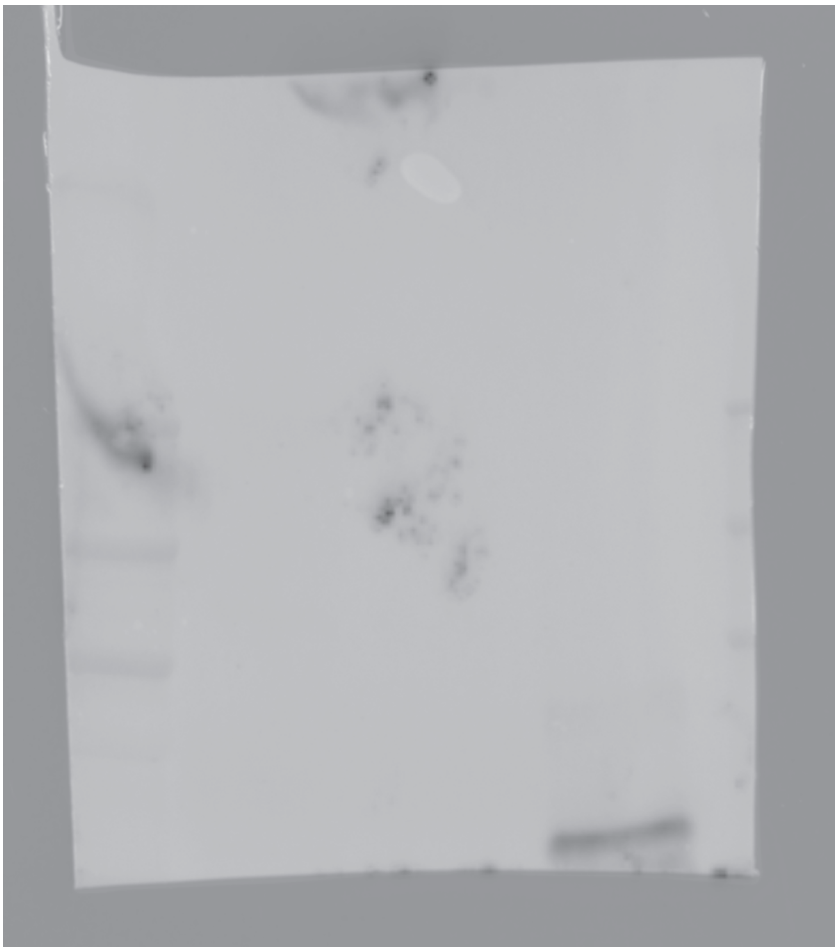

Figure 6A. Uncropped raw images

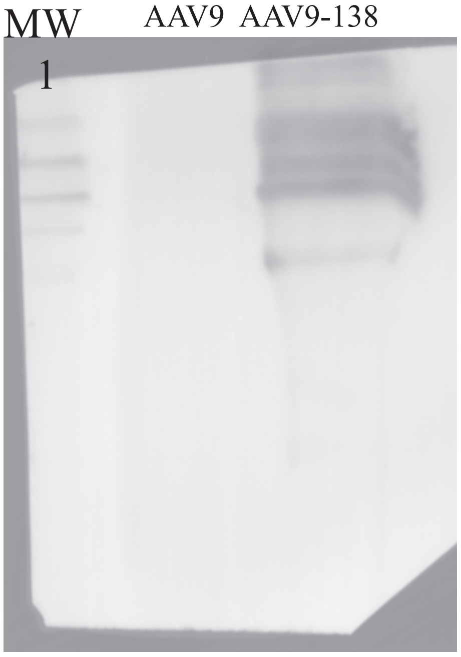

HDAC4

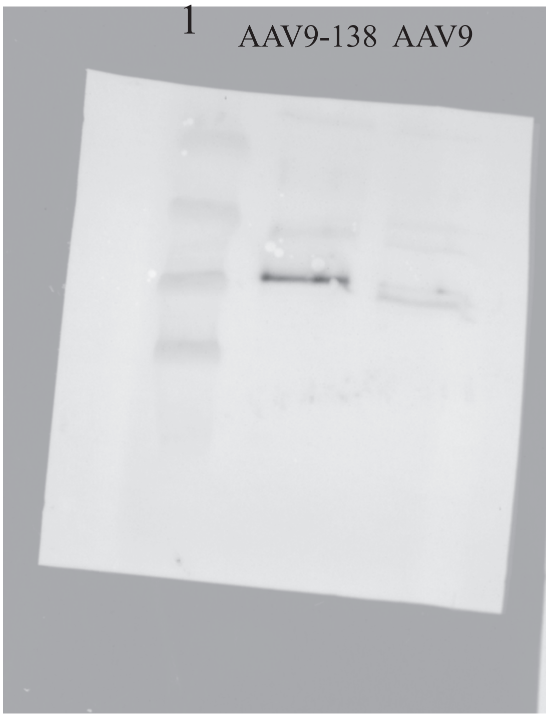

ALYREF

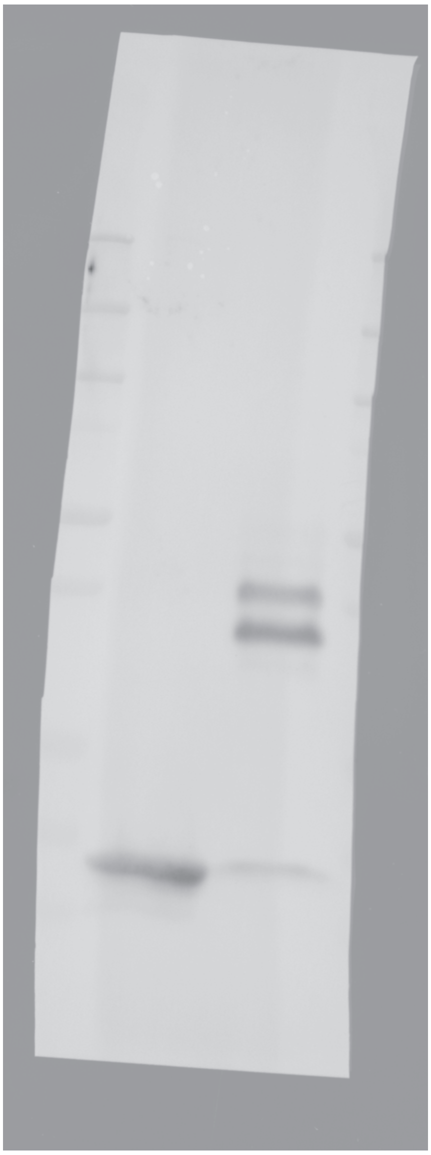

hnRNPA1

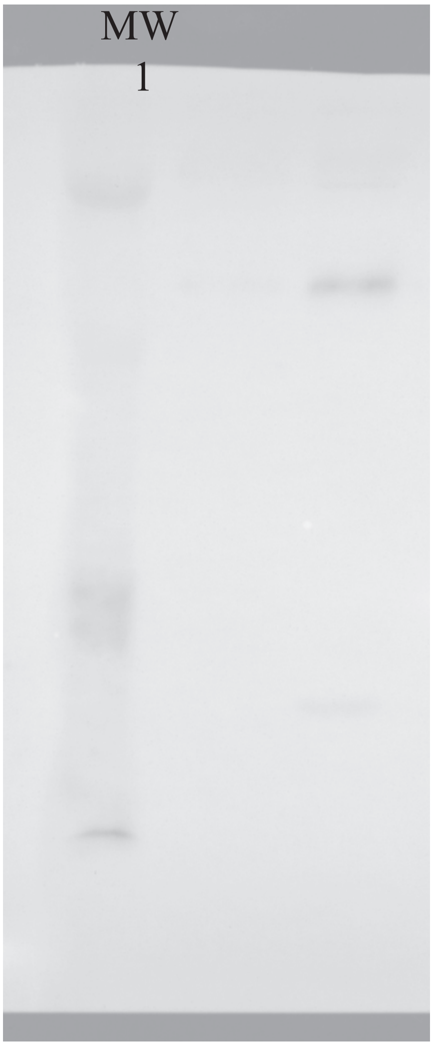

HDAC4

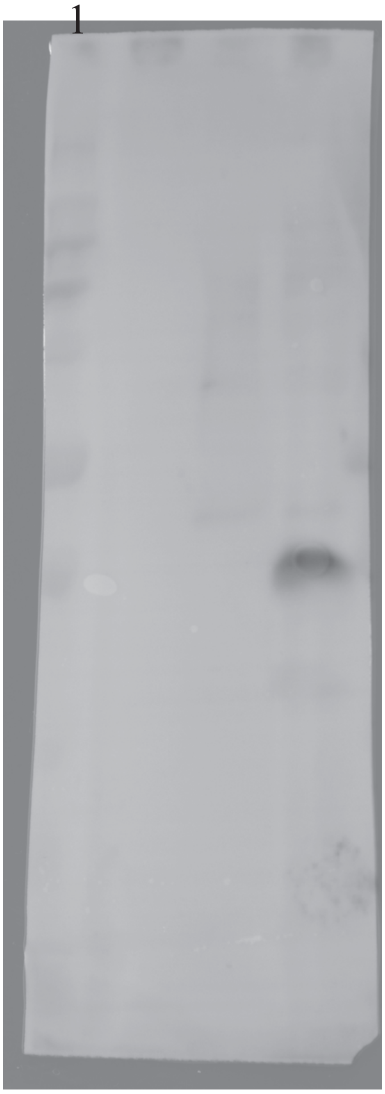

SRSF1

Figure 6B. Uncropped raw images

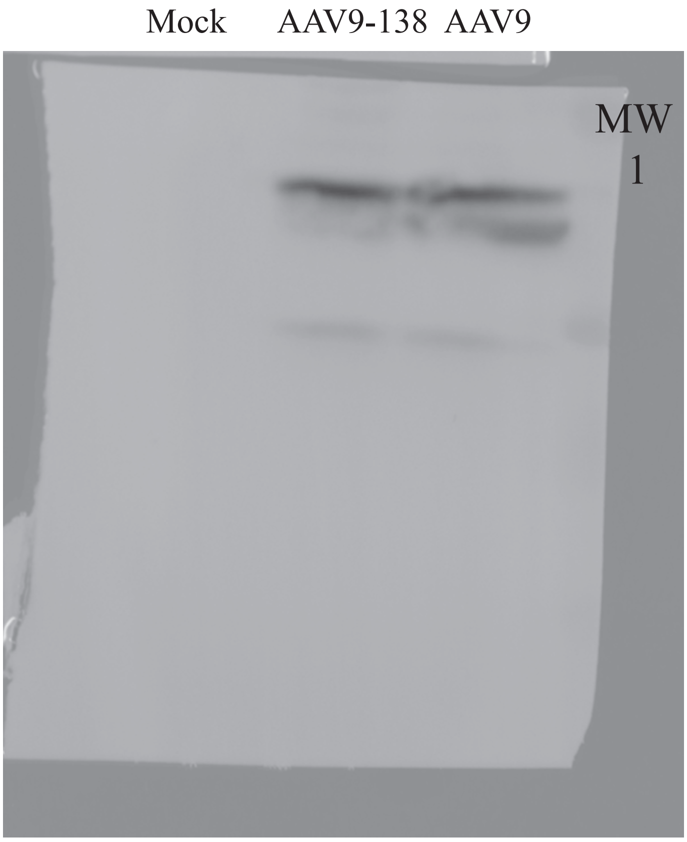

AAV

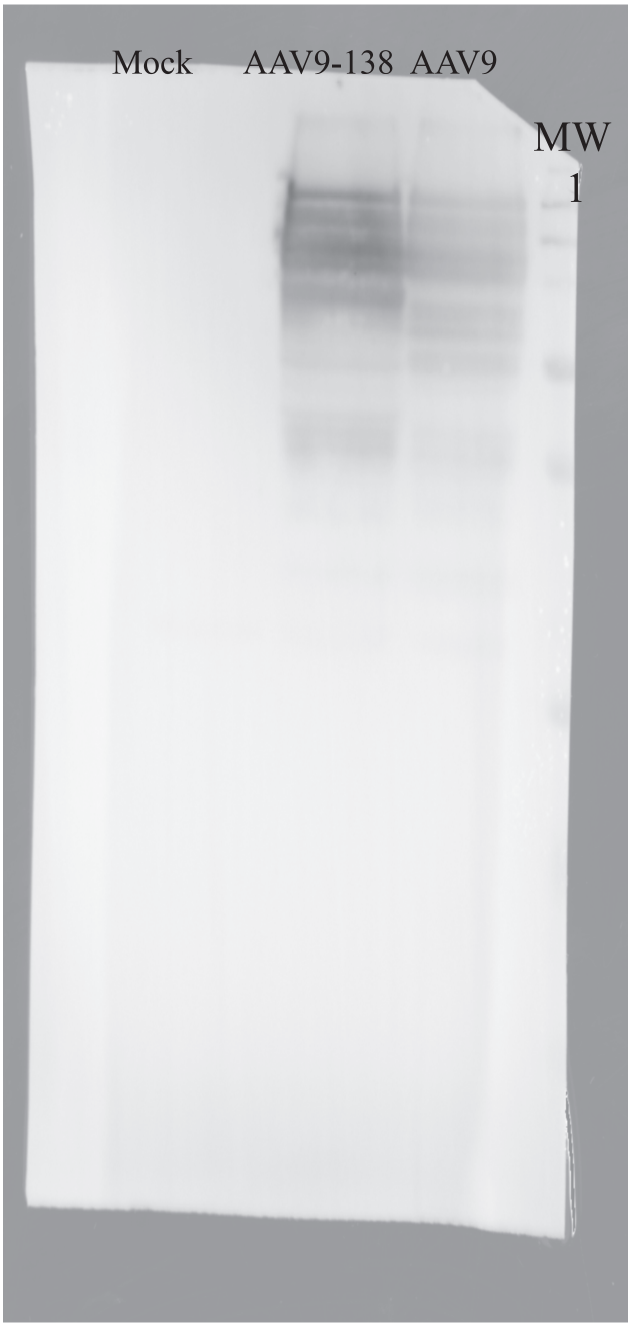

HDAC4

Figure 6D. Uncropped images

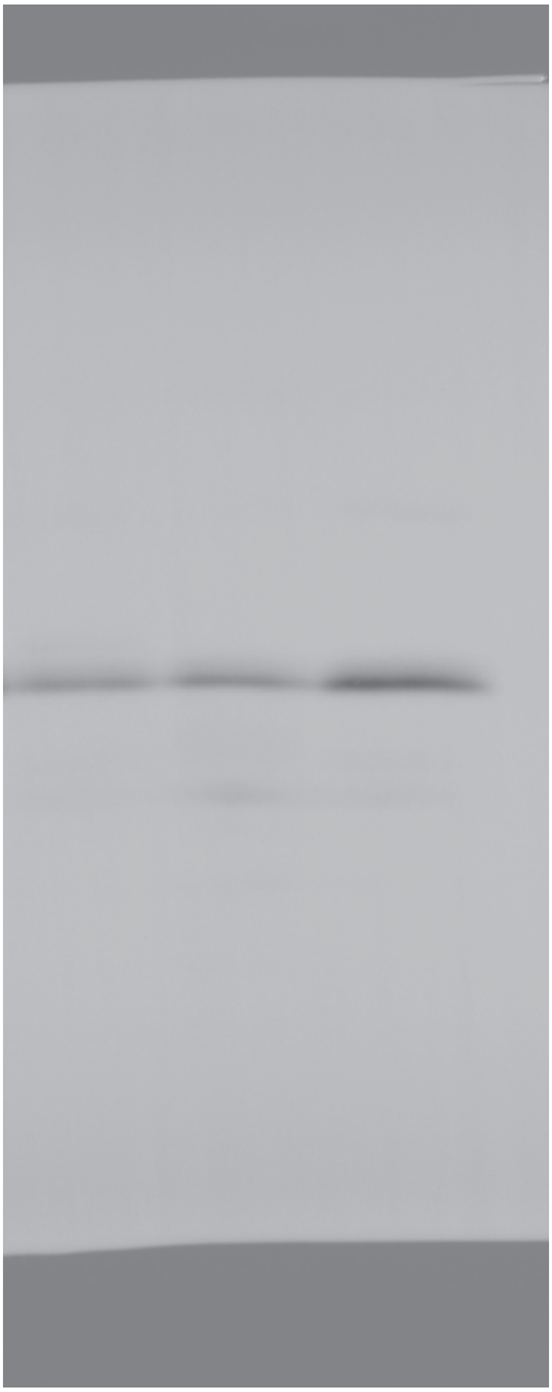

mcherry

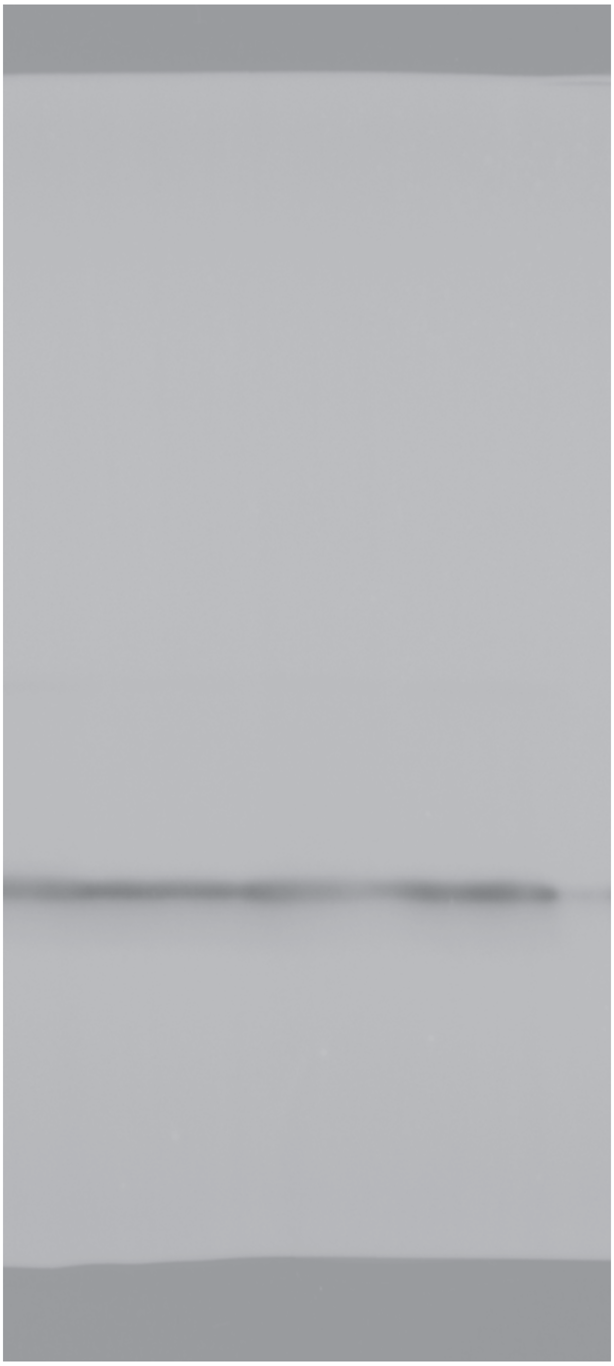

mtubulin
